# Narcissism as an Adaptive Trait for Successful Migration

**DOI:** 10.1177/14747049261445049

**Published:** 2026-04-21

**Authors:** Lauren O’Rourke, Rose McDermott, Pete Hatemi

**Affiliations:** 1 242728 School of Government and Public Policy, University of Arizona, Tucson, USA; 2 6752 Department of Political Science, Brown University, Providence, USA; 3 8082 Department of Political Science, Co-Fund Microbiology, Penn State University, University Park, USA

**Keywords:** narcissism, migration, NPI, FFNI

## Abstract

Narcissism is viewed as both a heritable trait and an activated state, but little research has explored how narcissism manifests in populations from an evolutionary perspective. Relying on two nationally representative US samples, we explore how narcissism may be mobilized or manifest in populations that undergo major environmental changes such as migration. More specifically, we treat immigrant generational status as a naturally occurring experiment and explore the differing levels of narcissism of new migrants, first, second and successive generations. We find new immigrants and first-generation Americans have the highest levels of narcissism, but those rates steadily decrease with later generations. Overall, narcissism appears to either be activated in populations under conditions of need, scarcity, stress, and survival, or be a key motivator for people who choose to migrate. Critically important, and from a generational perspective, narcissism declines to the new population level as migrants settle into their new environments.

There are many social, cultural, economic, and political factors that drive migration (Hatton & Williamson, 2002), and the topic has received a great deal of public concern and political debate over the last half century, especially in more economically developed nations. Yet, from an evolutionary perspective, the drivers of migration today are not dissimilar from those that humans have faced across millennia, including famine and war. Indeed, climate change, population pressures, and the need to find more food supplies, that is, scarcity and basic survival needs, are hypothesized as factors that drew the first humans out of Africa more than 80,000 years ago (Klein, 2008; Mellars, 2006). The human ability to move and migrate is believed to be critical for our survival and is partly responsible for humans achieving dominance over other species (Kuhn et al., 2016). In this way, migration has remained a ubiquitous evolutionary pressure, beginning even before the dawn of homo sapiens. However, there is much to learn about the individual psychological differences in migration patterns, including what personality factors are privileged in the motivation to migrate and the relative success or failure of individual migrants in today's world.

The social and environmental factors affecting migration are unlikely to be static; traits such as aggression, novelty-seeking, approach-avoidance motives, and many other factors are context dependent (Nikitin & Freund, 2019). Humans are remarkably adaptable and have both trait differences and state-based responses to various life pressures. One trait that remains understudied but emerges as a strong candidate likely to spur migration efforts, as well as differentially aid or hinder the success of such efforts, is narcissism. While not ignoring the social and environmental factors that contribute to migration (Hatton & Williamson, 2002), here we explore whether narcissism is differentially expressed in initial waves of migrant generations compared to those that become established in their new home across subsequent generations. In so doing, we consider the evolutionary role narcissism may have in driving the motivation to migrate, and how its role may change once populations become established in their new homeland.

We investigate narcissism as a population level survival trait, differentially emerging or activated under certain conditions of opportunity, stress and threat. The lay person may see narcissism as a wholly negative trait, and many may fall prey to, or for political or ideological purposes choose to, misinterpret findings that suggest that new migrants are higher in narcissism as an indication that immigrants are narcissists. That is not the purpose or prescription of this investigation, and such a view would be both untrue and unscholarly. We believe it is important to clarify this upfront. Instead, the personality trait of narcissism, not be confused with clinical narcissism, has both adaptive and maladaptive components, exists on a continuum, and is mostly normally distributed in the population (Campbell, 2001; Campbell et al., 2004). To some degree, narcissism is a necessary trait for all humans and serves to differentially regulate individual status needs, and facilitates the pursuit of material and reproductive outcomes (Back et al., 2013). All humans must have some level of self-interest to survive and function (Miller et al., 2011).

Narcissism is both a state and a trait (Luo & Cai, 2018) with differing individual baseline and population levels, both of which become upregulated or downregulated, depending on both person-specific and greater environmental conditions. Migration represents one of those momentous choices and actions that some people make and others do not, no matter the severity of their home country's situation. Those who choose to leave are different from the population who choose to stay under the same conditions; in this way, narcissism may operate as a motivating force for self-selection into migration. Thus, we examine narcissism as a means to explore how population traits are expressed differentially or change over time, and how such traits might become activated or diminished within a given population, whether under duress or because opportunity appears. Migration provides the ideal natural experiment for investigating the experience of such pressures or opportunities.

Grandiose narcissism, at its extreme, represents a personality trait marked by self-focus, entitlement, exploitation, a need for validation, confidence, dominance, antagonism and agentic extraversion (Miller et al., 2011). Deciding to move to a new country—a process that can involve having to learn a new language, cultural upheaval, social adjustment, identity reconstruction, social challenges, high risks, and personal reinvention—appears well-suited to both motivate or repress such traits and behaviors (Kreienkamp et al., 2024). Empirical research examining narcissism in relation to international relocation, however, remains limited. Indeed, we could not find any prior studies that directly examined the relationship between narcissism and mass migration. Existing studies tend to be anecdotal in nature (Gonzalez et al., 1999), or focus on the general personality traits of specific subsets of people, such as highly educated expatriates at the upper end of the economic spectrum, and how their personalities and abilities facilitate adjustment to their new career environment (Caligiuri, 2000; Fouarge et al., 2019). While studying these subgroups is important for corporate planning, career advancement, and the successful transition of employees, highly placed career-minded individuals represent only a small portion of migrants in the modern world, and do not reflect the mass migration patterns of today. This is not to say that there has been no clinical engagement on the matter. Indeed, a growing number of clinicians have recognized and spoken publicly about the suspected relationship between migrant generational status and narcissism (Cheng, 2025; Durvasula, 2023; Grannon, 2020; Kaur Kohli, 2025). Nevertheless, there remains an absence of nationally representative empirical studies on the topic.

In this paper, we explore the relationships between narcissism and immigrants’ generational status in two nationally representative US samples to examine the way in which environmental pressures or opportunities can serve to draw out, enhance or diminish certain trait characteristics. We begin with an overview of the proposed evolutionary role of narcissism, and the theoretical and operational properties of its psychological construct and sub-facets, seeking to understand the role of external circumstances on the population-level expression of the trait. We then develop our hypotheses and focus on the components of narcissism that manifest in migrants but appear to decline over generations as survival and other external pressures or opportunities diminish. Our analyses follow, treating immigrant generational status as a naturally occurring experiment where we demonstrate how narcissism decreases across successive immigrant generations. We conclude with a discussion of how narcissism either appears to motivate self-selection of individuals into migration or work as an adaptive trait for success (or both), but dissipates as migrants become generationally established.

## Grandiose Narcissism: An Evolved Activated State and Dispositional Trait

Narcissism is more than self-centeredness; it is a complex interrelated multidimensional set of traits, dispositions, and cognitive styles that motivative, regulate, and guide behaviors in a strategic manner. Narcissism represents both an inherited suite of traits, as well as a learned or activated state, with genetic and environmental underpinnings (Petrides et al., 2011) found in humans and other primates (Karterud, 2010). Narcissism is believed to result, at least in part, as a set of adaptations for social manipulation that foster short-term mating and social success with increased fitness through improved access to resources, survival, and reproductive opportunities (Holtzman & Donnellan, 2015). This facilitation occurs through advantages in social dominance, flexible self-promotion in competitive settings, and enhanced tactics for navigating hierarchical social environments to improve one's status (Gutiérrez & Valdesoiro, 2023; Volk et al., 2012). Overall, narcissism appears to be an adaptive trait-based and state-context-dependent suite of behaviors that balances benefits in short-term status with potential trade-offs in long-term relational instability (Lukaszewski et al., 2020).

Grandiose narcissism has agentic and antagonistic features that can result in positive or negative outcomes depending on the situation. Those higher in grandiose narcissism are more extraverted, agentic, sociable, and confident, with higher self-esteem, lower social anxiety, and greater approach orientations (Campbell et al., 2005; Rhodewalt & Morf, 1995). Individuals at the higher end of this trait desire authority and power. They are more self-sufficient, or at least have a greater belief in their own ability to meet their needs and achieve their goals (Barry et al., 2003). These agentic dimensions of narcissism, labeled Grandiose-Exhibitionism in the Narcissistic Personality Inventory (NPI), the most commonly used measure of narcissism (Raskin & Hall, 1981), reflect both social potency and emotional resilience (Ackerman et al., 2011).

The antagonistic facets of narcissism include aggressive, assertive, entitled, manipulative, and nonempathic behaviors. This side of grandiose narcissism, labeled Entitlement-Exploitation in the NPI, includes the belief that one is better, more superior, and more deserving than others, including expectations for special treatment and a demand that their needs be met. Their fantasies of glory and need for attention mix a desire for power and status with a belief in their right to manipulate and exploit others for their own personal gain. They have a need to win and dominate others and thus engage in display behaviors designed to gain followers or the admiration of others, in part to feed their vanity and in part to improve their status.

## Why Would Narcissism Manifest Differently by Immigrant Generation?

Two forces intersect in the decision to migrate: internally driven self-selection of those who choose to migrate as opposed to remaining in place; and the environmental pressures or opportunities encouraging migration (e.g., war, economic collapse, or persecution vs status gains, economic opportunity) that might upregulate narcissistic tendencies. In this way, both trait and trigger should have a role in differentially attracting those higher in narcissism and the expression of narcissism in individuals who decide to migrate. Again, this does not mean migrants are narcissists, rather we suggest an adaptationist rationale, whereby the incentives, both social and financial, created by the opportunity for migration, ranging from basic needs for survival to a potential increase in social status, better employment, or access to better social networks, push the inherent self-evaluation mechanisms in those higher in latent dispositional narcissism toward greater self-aggrandizement and enhanced self-focus. In other words, individuals higher in antagonistic forms of narcissism should be more likely to view migration as a viable strategy for improvement. In this way, they should be more likely to self-select into migration than those lower in the trait, given the same global environmental constraints and opportunities. Note that this expectation aligns with life-history and niche-selection models as well.

We operationalize our two factors in seeking to explore the relationship between narcissism and migrant generational status as Motivation and Adaptation. First, the *Motivation* to leave one's homeland to pursue a better life represents an audacious choice, reflecting the self-selection of individuals into migration. Even in the face of enormous pressures and terrible struggles, including life or death circumstances, only some people choose to leave, while others choose to stay. The smallest threat mobilizes some to leave their home country, often at great personal cost, while others do not see the same perceived threat in the same situation. On the other hand, a given threat may be over-perceived, and migrants may suffer more harm than good by leaving. Countless reports exist of those who died in the Darien Gap or along the southern U.S. border in high heat when migrating to the U.S. or Canada from Central and South America (Roy & Baumgartner, 2024). When the threat is real or greater than the risk of the journey itself, however, those who recognize it early may ensure their very survival. One need look no farther than the Jews who left Germany at the very outset of the Nazis’ rise to power as one example whereby early recognition of the threat and the willingness to incur the costs of leaving not only saved lives, but perhaps a culture and religion as well. Someone who chooses to leave everything behind with uncertain hopes for the future may indeed be desperate, but not only do people differentially perceive the world and its potential threats, but humans are also differentially confident or optimistic about their own ability to meet whatever challenges they may confront. Such individuals must believe they have different skills than those who decide to stay and often must put aside social norms to ensure their own success and survival. Furthermore, many may leave not because of survival, but in hopes of increasing personal wealth, or finding a mate, to satisfy their own ego drive, need for greatness or status, or to pursue a definition of success that rises above their peers’ wildest dreams.

Those higher in overall narcissism, and more specifically those higher on the narcissistic facets of Grandiose Exhibitionism (also labeled agentic extraversion), including its underlying self-absorption, self-promotion, superiority, admiration and status-seeking mechanisms, while potentially being motivated by economic, survival, or political factors, might also view moving to a new country as an opportunity to improve their status, and reinvent themselves or seek to assert dominance in a fresh social hierarchy. These individuals would be the ones who see themselves as better able to overcome the challenges of migration and be worthy of a new and potentially better life elsewhere. The aspect of grandiose narcissism that asserts agency and espouses extraversion, driven by a desire for rewards, might motivate such individuals to seek out the challenges of a new country as an opportunity for self-enhancement (Miller et al., 2021), and thus differentially self-select into migration. For instance, such individuals might leverage their confidence to network and pursue high-status opportunities in a new cultural context, perceiving the move as a chance to elevate their social and economic standing.

The Entitlement-Exploitation factor of narcissism (also labeled antagonistic narcissism) should also be higher in migrants and first-generation residents versus those of successive generations. This form of narcissism recognizes the shadow aspects of a characteristic that can increase short-term success but also cause long-term instability in social relations, as those who have been taken advantage of by such individuals avoid them in future interactions. Those higher in antagonistic narcissism are the ones who will do what it takes to make it, regardless of the personal or relationship costs to others. They have a strong belief in their right to do what they want or feel they must do. This combination of willingness to exploit others and a belief in the righteousness of their cause and actions are well suited to difficult and dangerous migration journeys. Those higher in entitlement and exploitation are also drawn to situations offering prestige or dominance. They strive to pursue status across various life transitions (Grapsas et al., 2020), suggesting that moving abroad, especially to a country perceived as offering better opportunities for economic or social advancement (e.g., from a developing nation to a more advanced industrial nation), is likely in part a result of this status-seeking drive. It is also important to consider the role that success has on narcissism in return. The act of migration, especially if one is successful in the new country, will likely potentiate higher degrees of narcissism, as individuals come to see themselves in an even better light in the wake of the obstacles they overcame and the success they achieve, as well as justifying their behaviors to overcome them in light of their subsequent success.

*Adaptation* is a second consideration and revolves around the fact that some traits are likely to make migrants more or less successful in adapting to new environments. The internal motivation to leave is a key facilitator in migration, but the traits that initiate the move may or may not necessarily be the same as those needed to fit well into a new environment. The traits that make moving in the first place possible may not prove beneficial for success, or in establishing stable life interactions once someone is ensconced in a new social, economic, and political milieu. Indeed, as populations become established, there is a well-documented tension between migrant, first, and subsequent generations’ views of themselves, their older generation family members, their ancestral culture, and their current world (Giguere et al., 2010; Stepick et al., 2001). The regulation and mitigation of narcissistic traits may play a vital role in migrants’ successful adjustment and self-perception over generations. Personality traits, including extraversion and openness, have been studied for their effect on expatriates’ ability to adapt to new cultural environments (Caligiuri, 2000). While there is little research in this area focusing on the interaction of narcissism and migration, a limited body of work finds that narcissistic individuals may struggle with cultural adjustment due to their self-centered focus, but paradoxically, they may also leverage their confidence to navigate unfamiliar settings effectively (Foster et al., 2003).

We can then expect that, as migrants become more settled, and their offspring become residents over generations at a population level, most narcissistic traits should decrease, including grandiose-exhibitionism and entitlement-exploitation factors because such characteristics are no longer as adaptive or necessary for survival or opportunity-taking. These are, or can become, suboptimal traits in many circumstances; for example, children higher in narcissism struggle to keep friends (Peets & Hodges, 2023). Recall, narcissism privileges short-term outcomes, often a cost to long-term relational stability. Generationally speaking, this means that overall, narcissism should be highest in recent migrants because such tendencies increase their motivation to migrate as well as facilitate their short-term success and survival. However, such characteristics should become less pronounced in successive generations as higher narcissistic traits are no longer necessary for survival and may instead become counterproductive. In other words, if high levels of narcissistic behavior continue at a population level in offspring, they would hinder long-term stability.

Those higher in narcissism can be charming and compelling, especially at first blush, helping them to facilitate initial social connections, even if those relationships strain over time in the face of narcissists’ self-absorption (Dufner et al., 2013). The confidence and extraversion aspects of grandiose narcissism ease one's initial social integration into new environments in part, because higher mental toughness, charm and confidence in the face of obstacles indirectly reduces anxiety, stress and depression (Papageorgiou et al., 2023). However, their sense of entitlement and exploitation results in friction over time and increasing instability in social relationships, particularly if they violate the dominant social norms in their new society (Bushman & Baumeister, 1998; Campbell et al., 2005; Sękowski et al., 2023). In this way, new environments may precipitate the socially triggered activation or deactivation of narcissism in and of itself. People must be able to adapt to their new circumstances, as well as find the mental wherewithal to overcome obstacles or defeat they encounter over the course of assimilation. However, these pressures reduce across each subsequent generation; that is, new environments should trigger population-level narcissism, but as those environments are successfully adapted to by successive generations, narcissism should decrease. This theoretical prospect has yet to be tested, as we do in the current study. Our expectation is that new migrants and first-generation migrants will be higher in both grandiose exhibitionism and entitlement and exploitation, but successive generations should look closer to the existing population in the new home country.

While we have strong reasons to propose that overall grandiose narcissism, as well as exhibitionism and antagonism, should differ by migrant generational status, other forms of narcissism do not appear as relevant for migration. Nevertheless, we include them in the analyses for completeness. Specifically, we have no expectations that the Leadership/Authority-seeking (NPI) facet of Grandiose Narcissism should differ by migrant generational status. Migration on its own does not offer a leadership role, nor does adapting to a new environment. These should differ naturally in all populations, but we have no expectation that it will be affected by migration on its own.

## Methods

### Study 1, 2021 Immigrant Generational Status and the Narcissistic Personality Inventory

Data was collected by YouGov between March 10–22, 2021; 1,100 respondents in the U.S. were matched to a nationally representative sampling frame on age, educational attainment, gender, race, and income. The frame was constructed by stratified sampling from the 2018 U.S. Census American Community Survey.

The dependent variable, Grandiose Narcissism, is measured by the Narcissistic Personality Inventory, the most widely used measure for the personality trait of narcissism (NPI, see Raskin & Hall, 1981). Forced-choice dyads ask respondents to choose between one of two opposing statements about themselves (e.g., “I insist upon getting the respect that is due me” vs. “I usually get the respect that I deserve”). Each is coded such that a “2” indicates the more narcissistic answer, and a “1” indicates the lower narcissistic answer. The NPI originally consisted of 40 questions that capture narcissism through seven facets (authority seeking, entitlement, exhibitionism, exploitativeness, self-sufficiency, superiority, and vanity). The measure has since been refined and reconceptualized to 25 items, and restructured to focus on internal consistency and improved theoretical validity, producing an overall Narcissism score (α = .82) and three subdimensions: Grandiose Exhibitionism (GE, α = .75); Entitlement/Exploitativeness (EE, α = .48); and Leadership/Authority (LA, α = .74) (Ackerman et al., 2011). All narcissism measures were standardized, and divided by two standard deviations to allow for easier interpretation and comparison (Gelman, 2008). This resulted in means of 0, with standard deviations of 0.5.

Our primary predictor of interest is generational status, which is coded Immigrant (1), First Generation (2), Second Generation (3), and Third Generation and onward (4). The original data separated out immigrant citizens and immigrant non-citizens, but we combined the two as they are theoretically the same for our hypotheses (empirically as well, please see the supplementary materials). Additional independent variables include a dichotomous measure of sex (1 for male); a six-item measure for education, (no high school to post-graduate education), age in years; a six-item measure for church attendance (never to more than once a week); and a 16-item measure for family income (< 10,000–500,000 +). Descriptive statistics for these variables are presented in Table 1, and raw frequencies are available in the supplementary information (SI).

**Table 1.** Demographic Information & Descriptive Statistics, 2021 Study.

NPI Variable	N	Mean	SD	Min	Max
Overall Narcissism	1071	0.00	0.50	−0.78	1.71
Grandiose Exhibitionism (GE)	1089	0.00	0.50	−0.50	1.75
Entitlement/Exploitativeness (EE)	1089	0.00	0.50	−0.37	1.69
Leadership/Authority Seeking (LA)	1084	0.00	0.50	−0.78	1.22
Immigrant Generational Status	1100	3.26	1.01	1.00	4.00
Male	1100	0.48	0.49	0.00	1.00
Education	1100	3.47	1.50	1.00	6.00
Age	1100	48.62	18.09	19.00	93.00
Church Attendance	1061	2.81	1.74	1.00	6.00
Family Income	957	6.19	3.63	1.00	16.00

### Study 2, 2023 Immigrant Generational Status and the Five-Factor Narcissism Inventory

Data was collected by YouGov between May 25 to June 1, 2023. YouGov interviewed 975 respondents, who were matched down to a sampling frame on age, education, sex, race, and political orientation (n = 870). The data are representative of the US based upon the American Community Survey (ACS), public voter file records, the 2020 Current Population Survey (CPS), Voting and Registration supplements, the 2020 National Election Pool (NEP) exit poll, and the 2020 presidential election. The primary independent variable, Immigrant Generational Status, is the same as in the 2021 data collection.

The 2023 study uses a newer, also validated and increasingly used alternative measure of narcissism, the 30-item Five-Factor Narcissism Inventory (FFNI, see West et al., 2021). The FFNI differs from the NPI in several ways. It includes both grandiose and vulnerable aspects of narcissism (NPI is only grandiose) and instead of using forced-choice dyads, the FFNI relies on Likert-type items (Strongly agree to Strongly disagree and coded such that a “1” indicates lower narcissism scores, and a “5” indicates higher scores). The FFNI contains 15 different subscales (2 items each): Acclaim-seeking; Arrogance; Authoritativeness; Distrust; Entitlement; Exhibitionism; Exploitativeness; Grandiose Fantasies; Indifference; Lack of Empathy; Manipulativeness; Need for Admiration; Reactive Anger; Shame; and Thrill-seeking. These facets can be combined in different ways to produce five conceptually distinct factors: an overall Grandiose narcissism score (acclaim-seeking, arrogance, authoritativeness, distrust, entitlement, exhibitionism, exploitativeness, grandiose fantasies, indifference, lack of empathy, manipulativeness, and thrill-seeking scales; α = .88); Agentic Extraversion (acclaim-seeking, authoritativeness, exhibitionism, and grandiose fantasies subscales; α = .77); Antagonistic narcissism (arrogance, distrust, entitlement, exploitativeness, lack of empathy, manipulativeness, reactive anger, and thrill-seeking subscales; α = .88); Neurotic narcissism (made up of the indifference (reverse coded), need for admiration, and shame subscales; α = .77); and Vulnerable narcissism (computed from the sum of the need for admiration, reactive anger, and shame scales; α = .75). We have no expectations that the Vulnerable or Neurotic (FFNI) facets of narcissism should differ by migrant generational status but include those measures for transparency and completeness in our analyses. Individuals higher on those traits, and those with ego or judgment sensitivity traits, should be no more or less motivated to, or successful at, migration; that is, such hypersensitivity to rejection could just as easily come from one's home country environment as from leaving one's home country environment.

In comparison, the FFNI Grandiose Narcissism measure would be equivalent to the NPI's overall composite score; Agentic Extraversion to the NPI's Grandiose Exhibitionism factor; and Antagonistic narcissism to the NPI's Entitlement/Exploitation factor. Descriptives for each composite are presented in Table 2, and correlations between measures can be found in the SI. Again, each measure was standardized to allow for better comparison between models (Gelman, 2008).

**Table 2.** Demographic Information & Descriptive Statistics, 2023 Study.

FFNI Variable	N	Mean	SD	Min	Max
Grandiose Narcissism	870	0.00	0.50	−1.26	1.88
Agentic Extraversion	870	0.00	0.50	−1.49	1.39
Antagonism	870	0.00	0.50	−1.15	1.98
Neurotic Narcissism	870	0.00	0.50	−1.24	1.37
Vulnerable Narcissism	870	0.00	0.50	−1.24	1.40
Immigrant Generational Status	870	3.29	1.00	1.00	4.00
Male	870	0.48	0.49	0.00	1.00
Education	870	3.47	1.52	1.00	6.00
Age	870	48.76	17.69	19.00	88.00
Church Attendance	844	2.78	1.74	1.00	6.00
Family Income	779	6.41	3.74	1.00	16.00

### Analyses & Results

**Figure 1.**
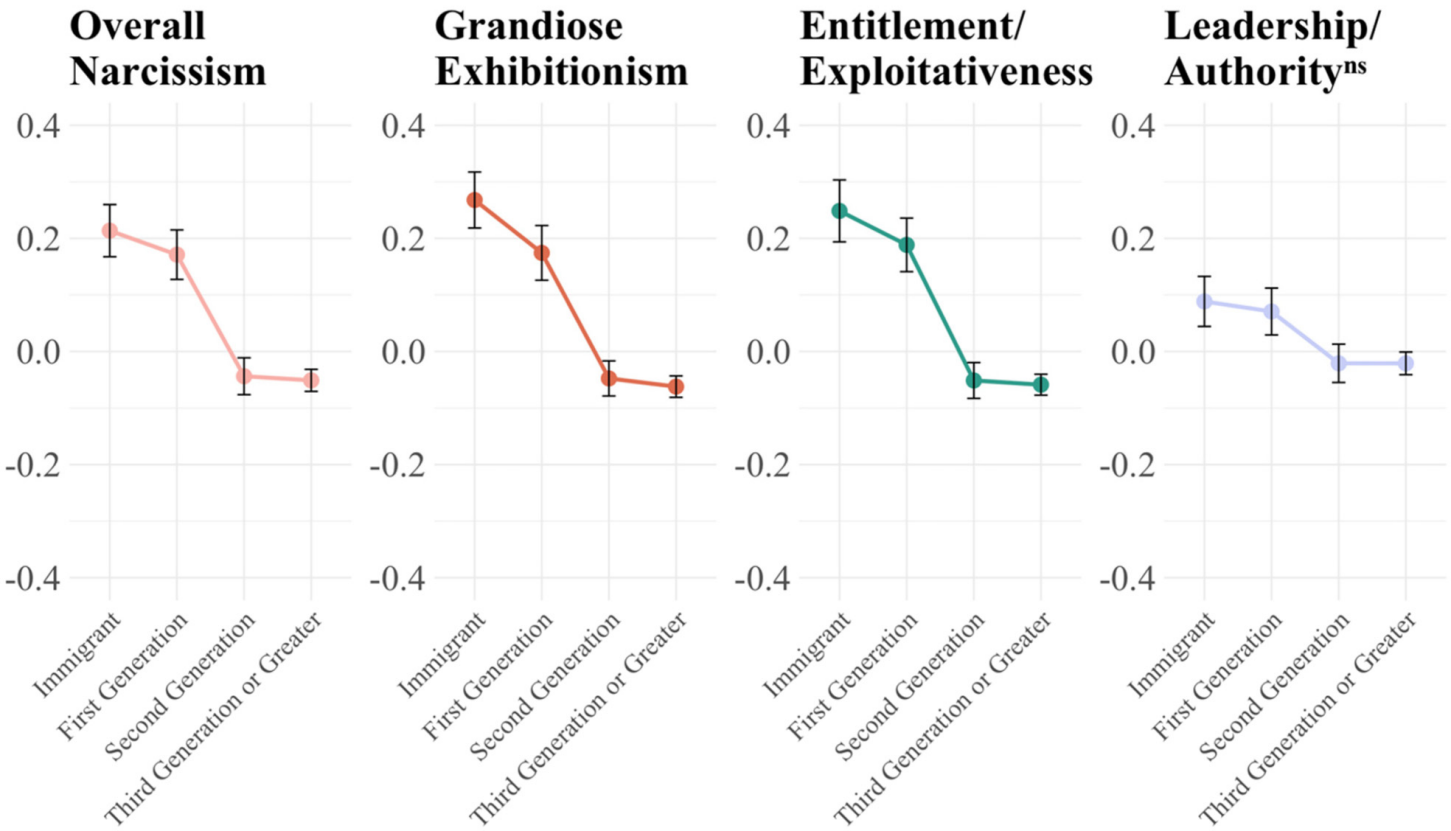
2021 NPI: narcissism declines as migrant generations become established. Notes: See Table 3 for significance test between generations. NS in superscript is not significant.

Analyses were conducted in R, version 2024.12.1 + 563. The data is deposited on the author's Dataverse (https://doi.org/10.7910/DVN/Y8OB0C). The core empirical finding of this study revolves around understanding whether and how rates of narcissism differ across successive immigrant generations. To best understand this, we compute ANOVA with Tukey's HSD analyses to identify which specific migrant generations, when compared with each other, are significantly different (correlations between measures are in the SI). We do this for each narcissism measure. The 2021 results are shown in Figure 1 & Table 3. Overall, we find support for our hypotheses that Narcissism and its subfactors of Grandiose Exhibitionism and Entitlement/Exploitativeness are significantly higher in migrants and first generations and steadily decline with later generations as they become more established. New migrants and first generations are not significantly different from one another, and the same holds true for later generations (2nd and 3rd) compared to themselves. We find no difference in the Leadership/Authority-Seeking facet, as expected.

**Table 3.** Mean Differences in Narcissism by Migrant Generational Status, 2021.

Generational Comparisons	Overall Narcissism	Grandiose Exhibitionism	Entitlement / Exploitativeness	Leadership / Authority Seeking
Immigrant vs. First	−0.04	−0.09	−0.06	−0.01
Immigrant vs. Second	−0.25***	−0.31***	−0.29***	−0.10
Immigrant vs. Third	−0.26***	−0.33***	−0.30***	−0.10
First vs. Second	−0.21***	−0.22***	−0.23***	−0.09
First vs. Third	−0.22***	−0.23***	−0.24***	−0.09
Second vs. Third	−0.00	−0.01	−0.00	−0.00

*p < .05, **p < .01, ***p < .001.

**Figure 2.**
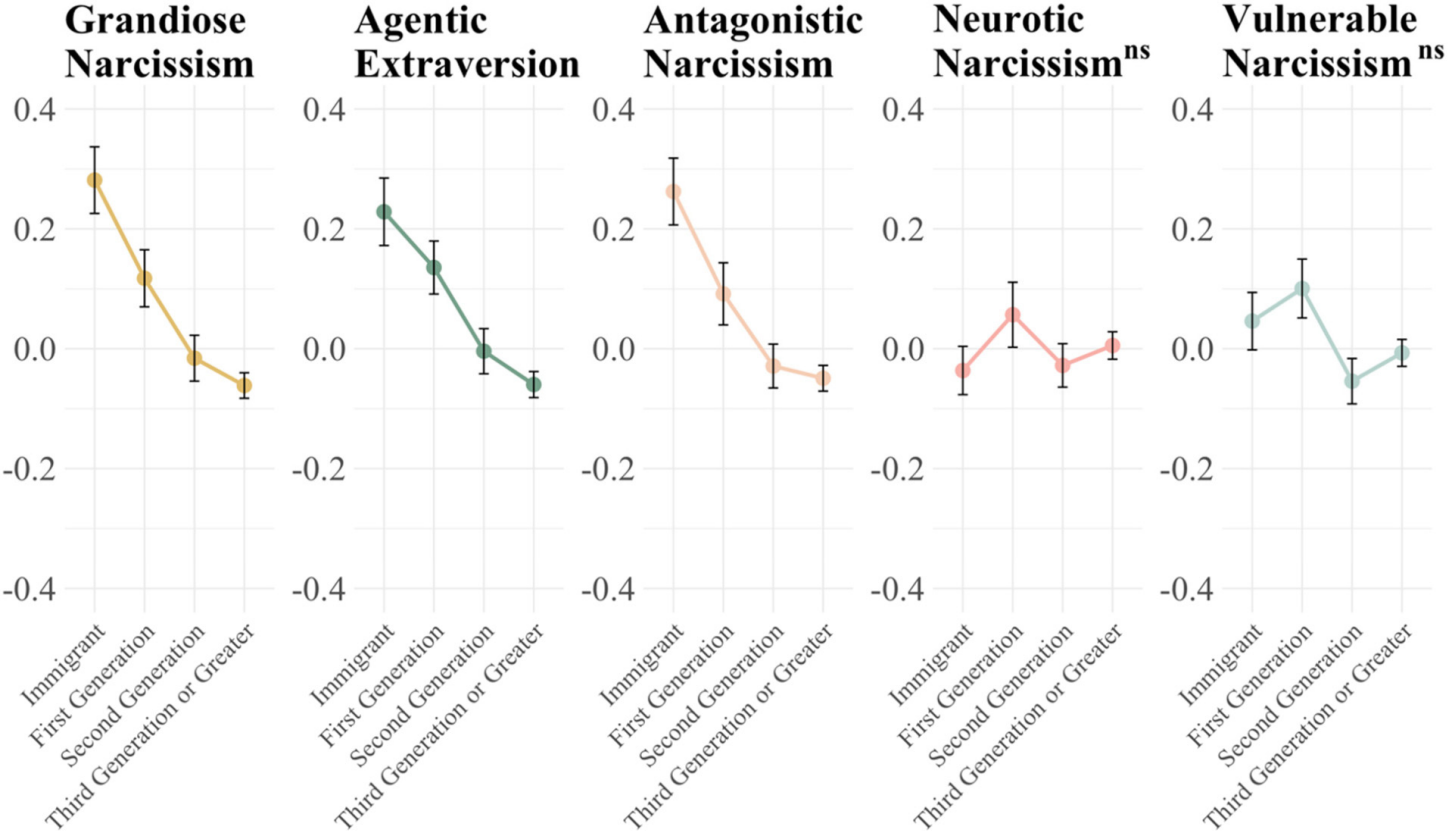
2023 FFNI: narcissism declines as migrant generations become established. Notes: See Table 4 for significance test between generations. NS in superscript is not significant.

Turning to the 2023 data, we run the same analyses (bivariate correlational analyses are reported in the SI). As before, the primary analyses are ANOVA and Tukey's post-hoc tests. The results are presented in Figure 2 and Table 4. Using the Five-Factor measure of narcissism, we find confirmatory evidence that new migrants and first-generation Americans have higher levels of Grandiose Narcissism, Agentic Extraversion, and Antagonistic narcissism compared to later generations. Again, we find no meaningful differences in the other subfacets of narcissism not theoretically related to migration motivations or success (Neurotic and Vulnerable narcissism, respectively).

**Table 4.** Mean Differences in Narcissism by Migrant Generational Status, 2023.

Generational Comparison	Grandiose Narcissism	Agentic Extraversion	Antagonism	Neurotic Narcissism	Vulnerable Narcissism
Immigrant vs. First	−0.16	−0.09	−0.17	0.09	0.05
Immigrant vs. Second	−0.29***	−0.23**	−0.29***	0.00	−0.10
Immigrant vs. Third	−0.34***	−0.28***	−0.31***	0.04	−0.05
First vs. Second	−0.13	−0.13	−0.12	−0.08	−0.15
First vs. Third	−0.17**	−0.19**	−0.14*	−0.05	−0.10
Second vs. Third	−0.04	−0.05	−0.02	0.03	0.04

*p < .05, **p < .01, ***p < .001.

To test the robustness of these findings, we conducted linear regression analyses with demographic predictors of narcissism, including age, sex, educational attainment, family income, and church attendance, on the NPI and FFNI measures. Migrant generational status remained a significant (and the largest) predictor for overall Narcissism, Grandiose Exhibitionism and Entitlement/Exploitation in the 2021 NPI data and Grandiose Narcissism, Agentic Extraversion, and Antagonism in the 2023 FFNI data.

## Limitations

The data have many strengths, including two nationally representative US samples, with two different, widely used, and validated measures of narcissism, the NPI and FFNI, and the power and measurement properties that provide confidence in our results. However, like all data, it has limitations. To begin, cultural context of both the home country and the new country and how they interact may trigger or depress narcissistic activation in unique ways. There is an active and unresolved debate on whether or how narcissism aligns more or less with individualistic cultures where self-promotion is often rewarded, versus collectivistic settings where social harmony is prioritized over individual assertion (Fatfouta et al., 2021; Foster et al., 2003). However, for the purpose of this study we need make no claim one way or the other, and critically important, the current study is a representative population-based study. That is, with two representative samples, we are capturing migrants and their offspring generations in a representative fashion to make a generalized conclusion, which is the aim of the study. Nevertheless, our results may only apply to migration to advanced liberal democracies in general or to the U.S., in particular.

Finally, the data can only contain information on who migrated and made it, including their offspring. This has two important implications for our results. First, we cannot directly compare the narcissism of those from the home country to those who left, and second and more importantly, we cannot compare the narcissism to those that made the journey where they and their offspring stayed in the US versus those who either perished on the journey or returned to their prior home after a failed attempt to relocate. It would be impossible to obtain such data, especially from those who are deceased, so some assumptions must be made. It may be, for example, that those with higher levels of narcissism are more likely to stay and succeed than those who return.

## Discussion

Narcissism, in both its agentic and antagonistic forms, is significantly different and highest in migrants and first-generation immigrants but decreases in subsequent migrant generations. These findings remain only a first step. With our data, we cannot pull apart the relative importance of the three potential pathways on why higher narcissism manifests in new migrant populations: 1) People who are higher in narcissism are the ones who choose to migrate; 2) People who are higher in narcissism are the ones successful in migrating; 3) People who migrate become more narcissistic. Indeed, narcissism's role at the individual level, regarding how people might be more or less likely to migrate or how narcissistic traits manifest due to stress, financial pressures, and the act of emigrating as a form of self-protection and survival are interdependent. In other words, it appears likely the relationship between narcissistic traits and migration is bidirectional. For example, achieving social prominence abroad might bolster grandiose tendencies, creating tension with later generations who do not need to manifest narcissistic traits to survive or succeed, and indeed might hinder their ability to establish stable relationships. In later generations, diminished narcissism may, in fact, potentiate greater success by allowing for more stability in social and mating relationships over time. This hypothesis aligns with plasticity theories, suggesting life transitions can alter personality traits across generations through genetic and environmental interaction (Bonduriansky, 2021; Conger et al., 2021; Roberts & Mroczek, 2008).

In this way, as with many other traits and effects, the outcomes we witness represent an interaction between a trait characteristic, in this case narcissism, and an environmental precipitant, in this case, migration. The stress and risk that incentivize migration may help to upregulate or trigger underlying narcissistic tendencies in those who have lower thresholds for threat, or whose desire for success raises their risk acceptance. Those higher in narcissism should be the ones who self-select into migration and succeed, but once in a new environment, tendencies that privilege narcissism may also lead to greater tension with the surrounding community over time, and even with their own descendants. Subsequent generations may not need to display higher self-interest or exploitative tendencies to fit in and succeed in the new environment, which is, of course, for them, not new, but rather all they have known.

Here we have identified the phenomena in terms of narcissism and migration in the modern world, but there is no reason to think that such processes are limited to the migration experience or to narcissism. Rather, we would expect that an underlying mechanism designed to maximize prospects for survival would privilege those behaviors that are more likely to lead to that outcome under given environmental constraints and circumstances. In the case of migration, threats to survival may help activate tendencies that increase the incentive to move as well as maximize the likelihood of success in novel environments. In our data, those strategies appear to include the facets of grandiose narcissism that protect an individual from paralysis or surrender and provide confidence in the face of enormous opposition and enhance the kind of social potency that increases the odds of success in a novel and potentially hostile new environment. Across subsequent generations, when that environment is no longer new or threatening, survival or success is more likely under conditions that rely on stable social relationships and the lower degree of narcissism that allows for successful child rearing, thus decreasing the necessity of the most exploitative aspects of grandiose narcissism and increasing the value of more cooperative forms of social engagement. Thus, successful adaptation to a new environment depends both on the latent characteristics of individuals as well as the environmental challenges and opportunities they encounter.

Alternative explanations for our findings exist of course. Including factors such as cultural assimilation, normative constraint, and regression toward the mean, while all part of evolution, have unique implications. If those who migrate derive differentially from the higher end of the narcissistic distribution, as we expect, then one can also expect that the subsequent population might return closer to the mean, as we find in our data. If this statistical regularity contributes to lower levels of narcissism in subsequent generations, then our findings may be most informative for understanding the motivation to self-select into migration and be less informative for the adaptation of those who follow. Additional research may be able to tease these possibilities apart, but because our data indicate that narcissism does not significantly decline until the second generation, this suggests that at least part of the decline results from the processes we suggest are operative.

Migration is an enormous and increasing global phenomena that has produced contentious political divisions, particularly within destination countries. Migration offers many potential benefits for those who migrate, as well as the host countries who may seek additional labor, but also comes with intrinsic challenges associated with assimilation. Increasing environmental pressures, such as widespread climate change effects including fires, floods, droughts and famine, or endemic violence, will only place more pressure on individuals in high-risk areas, increasing the drive for migration among those most willing to risk high costs for the possibility of large rewards. Understanding more about the kinds of people who choose to migrate, and why, along with the processes of assimilation over time, can help both countries experiencing high rates of out-migration, as well as those areas that have high levels of in-migration, better facilitate the challenges and opportunities posed by migration, including in the realm of integration, training and job placement, as well as support services. Many factors contribute to the decision to migrate, and narcissism appears to be an important contributor. Across generations, environmentally triggered activation may diminish, but such divergence can help explain the common experience of inter-generational misunderstanding and conflict. This factor alone poses enormous consequences if members of the younger generation are expected to bear the burden of caretaking for older relatives whose higher tendencies toward exploitativeness prove challenging.

## Supplemental Material

sj-docx-1-evp-10.1177_14747049261445049 - Supplemental material for Narcissism as an Adaptive Trait for Successful Migration Supplemental material, sj-docx-1-evp-10.1177_14747049261445049 for Narcissism as an Adaptive Trait for Successful Migration by Lauren O’Rourke, Rose McDermott and Pete Hatemi in Evolutionary Psychology
