# Supplementary material for: Narcissism as an Adaptive Trait for Successful Migration
Source: Evol Psychol. 2026 Apr 21;24(2):14747049261445049. doi: 10.1177/14747049261445049 (PMC13111831; doi:10.1177/14747049261445049)
Supplement: sj-docx-1-evp-10.1177_14747049261445049 - Supplemental material for Narcissism as an Adaptive Trait for Successful Migration [file sj-docx-1-evp-10.1177_14747049261445049.docx]

Online Supplementary Materials

Narcissism as an Adaptive Trait for Successful Migration

**SI1: Raw Variable Measurement, 2021 & 2023**

**2021 NPI Factors** (Ackerman, 2011)

NPI1: I have a natural talent for influencing people vs. I am not good at influencing people (Leadership/Authority Seeking)

NPI2: When people compliment me I sometimes get embarrassed vs. I know that I am good because everybody keeps telling me so (Grandiose Exhibitionism)

NPI3: The thought of ruling the world frightens the hell out of me vs. If I ruled the world it would be a better place (Leadership/Authority Seeking)

NPI4: I prefer to blend in with the crowd vs. I like to be the center of attention (Grandiose Exhibitionism)

NPI5: I am not sure if I would make a good leader vs. I see myself as a good leader (Leadership/Authority Seeking)

NPI6: I am assertive vs. I wish I were more assertive (Leadership/Authority Seeking)

NPI7: I like to have authority over other people vs. I don't mind following orders (Leadership/Authority Seeking)

NPI8: I find it easy to manipulate people vs. I don't like it when I find myself manipulating people (Entitlement/Exploitativeness)

NPI9: I insist upon getting the respect that is due me vs. I usually get the respect that I deserve (Entitlement/Exploitativeness)

NPI10: I don't particularly like to show off my body vs. I like to show off my body (Grandiose Exhibitionism)

NPI11: My body is nothing special vs. I like to look at my body (Grandiose Exhibitionism)

NPI12: I try not to be a show off vs. I will usually show off if I get the chance (Grandiose Exhibitionism)

NPI13: I expect a great deal from other people vs. I like to do things for other people (Entitlement/Exploitativeness)

NPI14: I will never be satisfied until I get all that I deserve vs. I take my satisfactions as they come (Entitlement/Exploitativeness)

NPI15: Compliments embarrass me vs. I like to be complimented (Grandiose Exhibitionism)

NPI16: I have a strong will to power vs. Power for its own sake doesn't interest me (Leadership/Authority Seeking)

NPI17: I don't care about new fads and fashions vs. I like to start new fads and fashions (Grandiose Exhibitionism)

NPI18: I like to look at myself in the mirror vs. I am not particularly interested in looking at myself in the mirror (Grandiose Exhibitionism)

NPI19: I really like to be the center of attention vs. It makes me uncomfortable to be the center of attention (Grandiose Exhibitionism)

NPI20: Being an authority doesn't mean that much to me vs. People always seem to recognize my authority (Leadership/Authority Seeking)

NPI21: I would prefer to be a leader vs. It makes little difference to me whether I am a leader or not (Leadership/Authority Seeking)

NPI22: I am going to be a great person vs. I hope I am going to be successful (Leadership/Authority Seeking)

NPI23: I am a born leader vs. Leadership is a quality that takes a long time to develop (Leadership/Authority Seeking)

NPI24: I get upset when people don't notice how I look when I go out in public vs. I don't mind blending into the crowd when I go out in public (Grandiose Exhibitionism)

NPI25: I am much like everybody else vs. I am an extraordinary person (Leadership/Authority Seeking)

**2023 FFNI Measures** (West et al. 2021)

FFNI1: When someone does something nice for me, I wonder what they want from me. (Grandiose Narcissism)

FFNI2: When people judge me, I just don't care. (Grandiose Narcissism & Neurotic Narcissism; reverse coded)

FFNI3: I don't worry about others' needs. (Grandiose Narcissism & Antagonism)

FFNI4: I'm pretty good at manipulating people. (Grandiose Narcissism & Antagonism)

FFNI5: I hate being criticized so much that I can't control my temper when it happens. (Vulnerable Narcissism & Antagonism)

FFNI6: I will try almost anything to get my "thrills". (Grandiose Narcissism & Antagonism)

FFNI7: I am comfortable taking on positions of authority. (Grandiose Narcissism & Agentic Extraversion)

FFNI8: I often fantasize about having lots of success and power. (Grandiose Narcissism & Agentic Extraversion)

FFNI9: I aspire for greatness. (Grandiose Narcissism & Agentic Extraversion)

FFNI10: I do not waste my time hanging out with people who are beneath me. (Grandiose Narcissism & Antagonism)

FFNI11: It may seem unfair, but I deserve extra (i.e., attention, privileges, rewards). (Grandiose Narcissism & Antagonism)

FFNI12: I feel ashamed when people judge me. (Vulnerable Narcissism & Neurotic Narcissism)

FFNI13: I love to entertain people. (Grandiose Narcissism & Agentic Extraversion)

FFNI14: I'm willing to exploit others to further my own goals. (Grandiose Narcissism & Antagonism)

FFNI15: I wish I didn't care so much about what others think of me. (Vulnerable Narcissism & Neurotic Narcissism)

FFNI16: I often think that others aren't telling me the whole truth. (Grandiose Narcissism & Antagonism)

FFNI17: I don't generally pay much attention to the woes of others. (Grandiose Narcissism & Antagonism)

FFNI18: I can talk my way into and out of anything. (Grandiose Narcissism & Antagonism)

FFNI19: I feel enraged when people disrespect me. (Vulnerable Narcissism & Antagonism)

FFNI20: I am a bit of a daredevil. (Grandiose Narcissism & Antagonism)

FFNI21: Others say I brag too much, but everything I say is true. (Grandiose Narcissism & Antagonism)

FFNI22: I believe I am entitled to special accommodations. (Grandiose Narcissism & Antagonism)

FFNI23: Sometimes to succeed you need to use other people. (Grandiose Narcissism & Antagonism)

FFNI24: I tend to take charge of most situations. (Grandiose Narcissism & Agentic Extraversion)

FFNI25: I often fantasize about someday being famous. (Grandiose Narcissism & Agentic Extraversion)

FFNI26: I am driven to succeed. (Grandiose Narcissism & Agentic Extraversion)

FFNI27: I like being noticed by others. (Grandiose Narcissism & Agentic Extraversion)

FFNI28: Others' opinions of me are of little concern to me. (Grandiose Narcissism & Neurotic Narcissism; reverse coded)

FFNI29: I feel foolish when I make a mistake in front of others. (Vulnerable Narcissism & Neurotic Narcissism)

FFNI30: I often feel as if I need compliments from others in order to be sure of myself. (Vulnerable Narcissism & Neurotic Narcissism)

**Immigrant & Generational Status Raw Categories, 2021 & 2023**

Immigrant citizen (1)

Immigrant non-citizen (2)

First Generation (3)

Second Generation (4)

Third Generation (5)

**Gender, 2021 & 2023**

Male (1)

Female (2)

**Education, 2021 & 2023**

No High School (1)

High School Graduate (2)

Some College (3)

2-year (4)

4-year (5)

Post-grad (6)

**Age, 2021 & 2023**

Calculated in both datasets by subtracting the birth year from the data collection year (2021 or 2023), resulting in age in years.

**Church Attendance, 2021 & 2023**

More than Once a Week (1)

Once a Week (2)

Once or Twice a Month (3)

A Few Times a Year (4)

Seldom (5)

Never (6)

Don’t Know (7)

**Family Income, 2021 & 2023**

< $10,000 (1)

$10,000 - $19,999 (2)

$20,000 – 29,999 (3)

$30,000 - $39,999 (4)

$40,000 - $49,000 (5)

$50,000 - $59,999 (6)

$60,000 - $69,000 (7)

$70,000 - $79,999 (8)

$80,000 - $99,999 (9)

$100,000 - $119,999 (10)

$120,000 - $149,999 (11)

$150,000 - $199,999 (12)

$200,000 - $249,999 (13)

$250,000 - $349,999 (14)

$350,000 - $499,999 (15)

$500,000 or more (16)

**SI2: Demographic and Narcissism Frequency Distributions**

**SI2.1:** 2021 Raw Data Demographic Frequencies

| **Variable** |  | **N** | **Mean** | **SD** | **Min** | **Max** |
| --- | --- | --- | --- | --- | --- | --- |
| Overall Narcissism | | 1071 | 32.22 | 4.61 | 25.00 | 48.00 |
| Grandiose Exhibitionism | | 1089 | 12.24 | 2.21 | 10.00 | 20.00 |
| Entitlement/Exploitativeness | | 1089 | 4.72 | 0.96 | 4.00 | 8.00 |
| Leadership/Authority Seeking | | 1084 | 15.30 | 2.74 | 11.00 | 22.00 |
| Immigrant Generational Status |  | 1100 | 4.19 | 1.18 | 1.00 | 5.00 |
| Gender |  | 1100 | 1.51 | 0.49 | 1.00 | 2.00 |
| Educational Status |  | 1100 | 3.47 | 1.50 | 1.00 | 6.00 |
| Age |  | 1100 | 48.62 | 18.09 | 19.00 | 93.00 |
| Church Attendance |  | 1100 | 4.28 | 1.79 | 1.00 | 7.00 |
| Family Income |  | 957 | 6.19 | 3.63 | 1.00 | 16.00 |
|  |  |  |  |  |  |  |

**SI2.2:** 2023 Raw Data Demographic Frequencies

| **Variable** |  | **N** | **Mean** | **SD** | **Min** | **Max** |
| --- | --- | --- | --- | --- | --- | --- |
| Grandiose Narcissism | | 870 | 64.17 | 12.66 | 32.00 | 112.00 |
| Agentic Extraversion | | 870 | 24.55 | 5.51 | 8.00 | 40.00 |
| Antagonism | | 870 | 39.50 | 10.20 | 16.00 | 80.00 |
| Neurotic Narcissism | | 870 | 17.40 | 4.57 | 6.00 | 30.00 |
| Vulnerable Narcissism | | 870 | 17.29 | 4.51 | 6.00 | 30.00 |
| Immigrant Generational Status |  | 870 | 4.22 | 1.18 | 1.00 | 5.00 |
| Gender |  | 870 | 1.51 | 0.49 | 1.00 | 2.00 |
| Educational Status |  | 870 | 3.47 | 1.52 | 1.00 | 6.00 |
| Age |  | 870 | 48.76 | 17.69 | 19.00 | 88.00 |
| Church Attendance |  | 870 | 4.30 | 1.78 | 1.00 | 7.00 |
| Family Income |  | 779 | 6.41 | 3.74 | 1.00 | 16.00 |
|  |  |  |  |  |  |  |

**SI2.3:** 2021 Raw Frequency Distributions


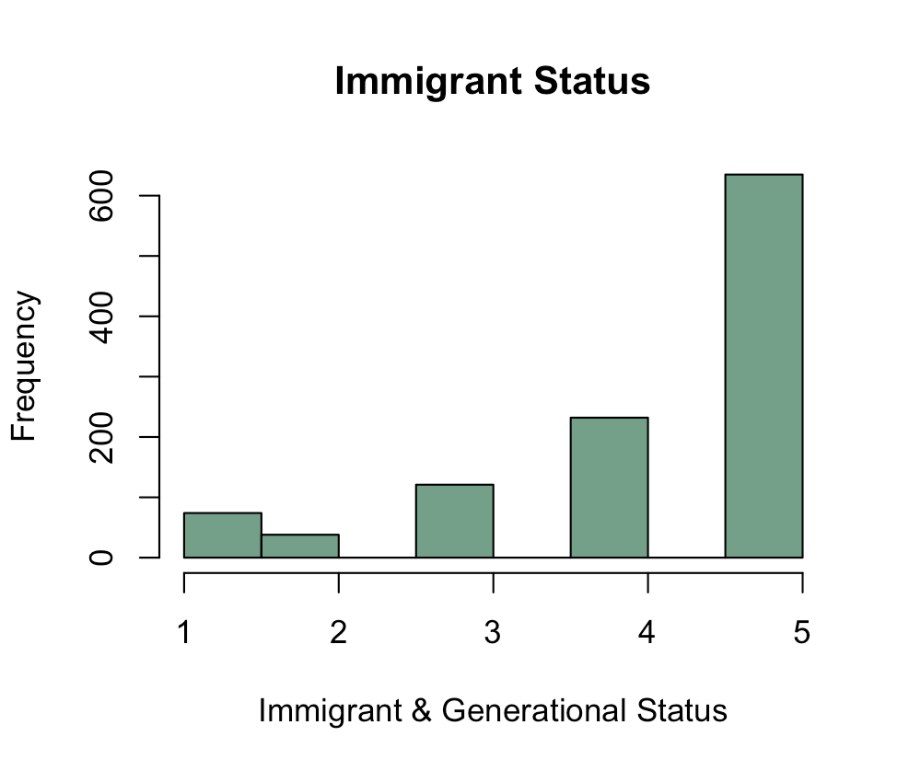


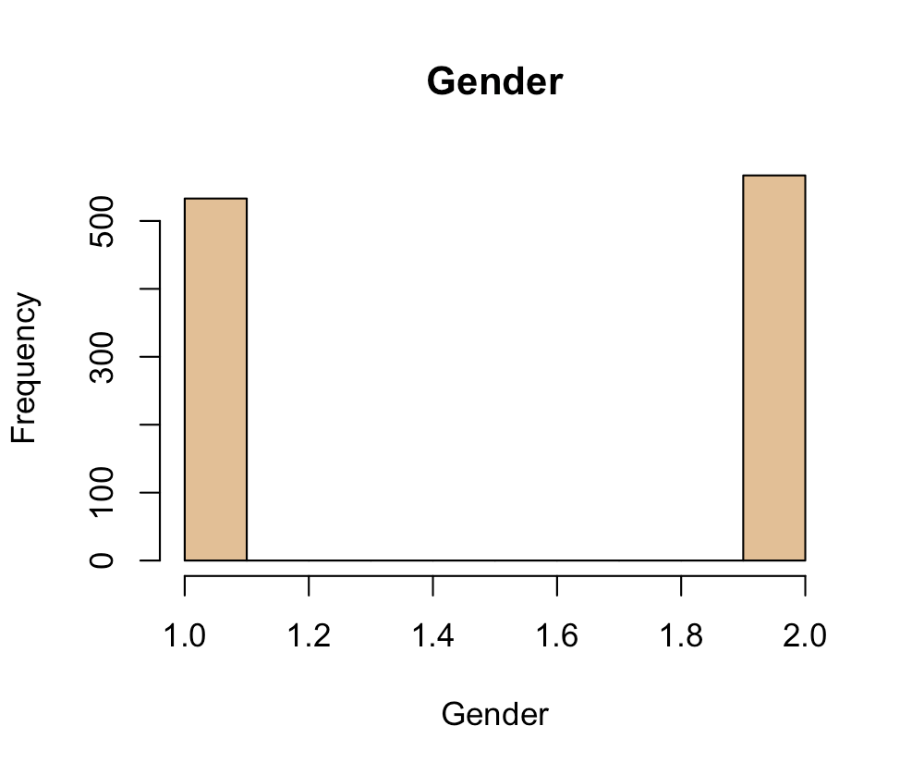


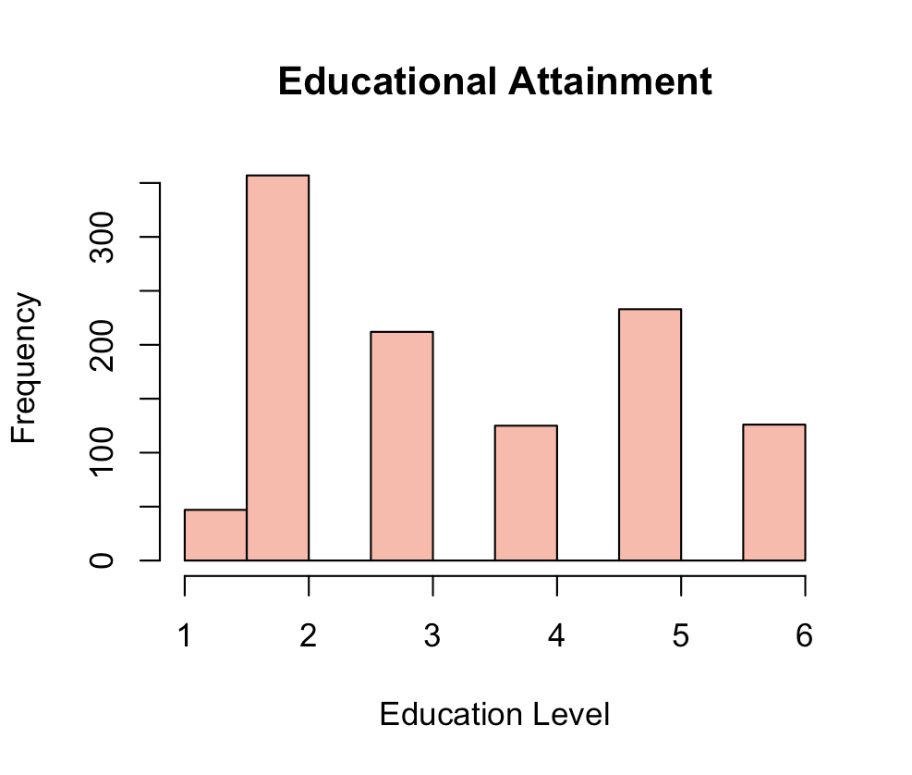


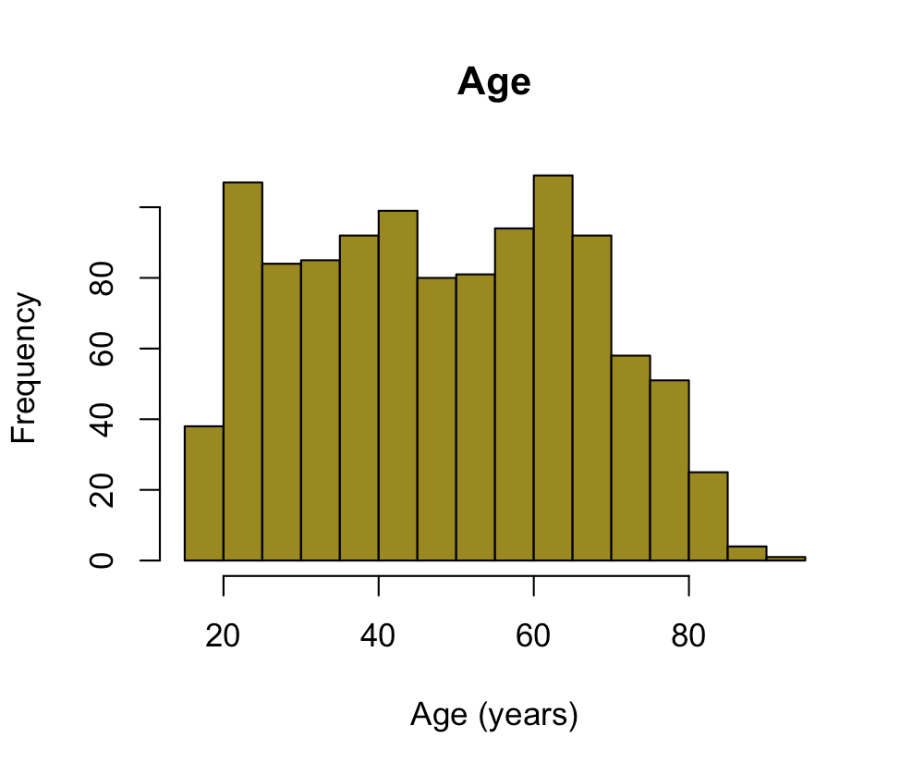


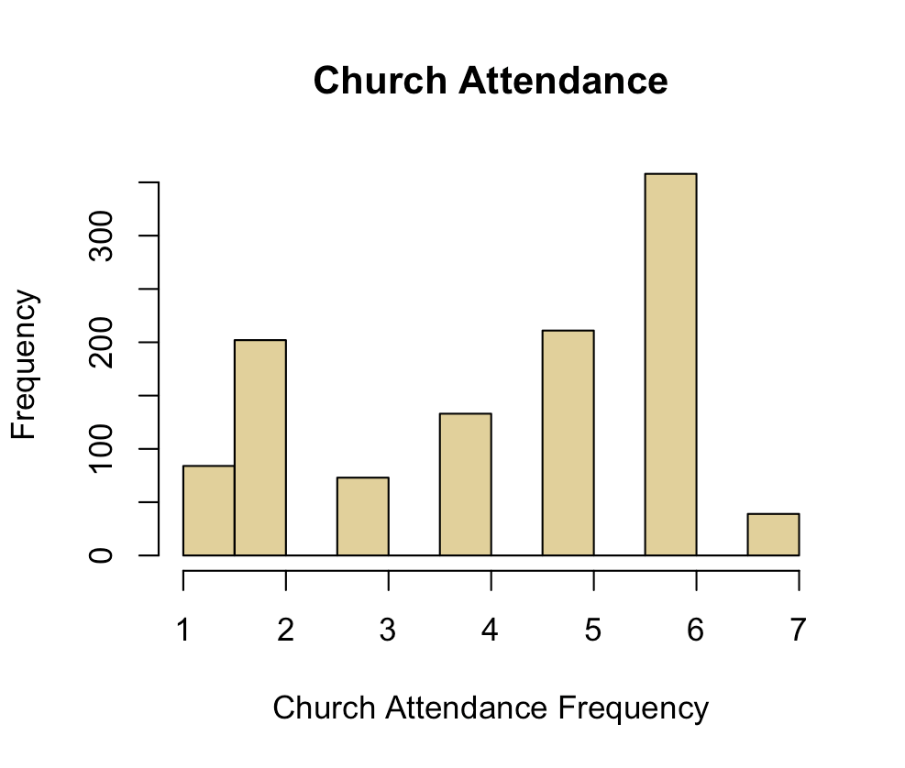


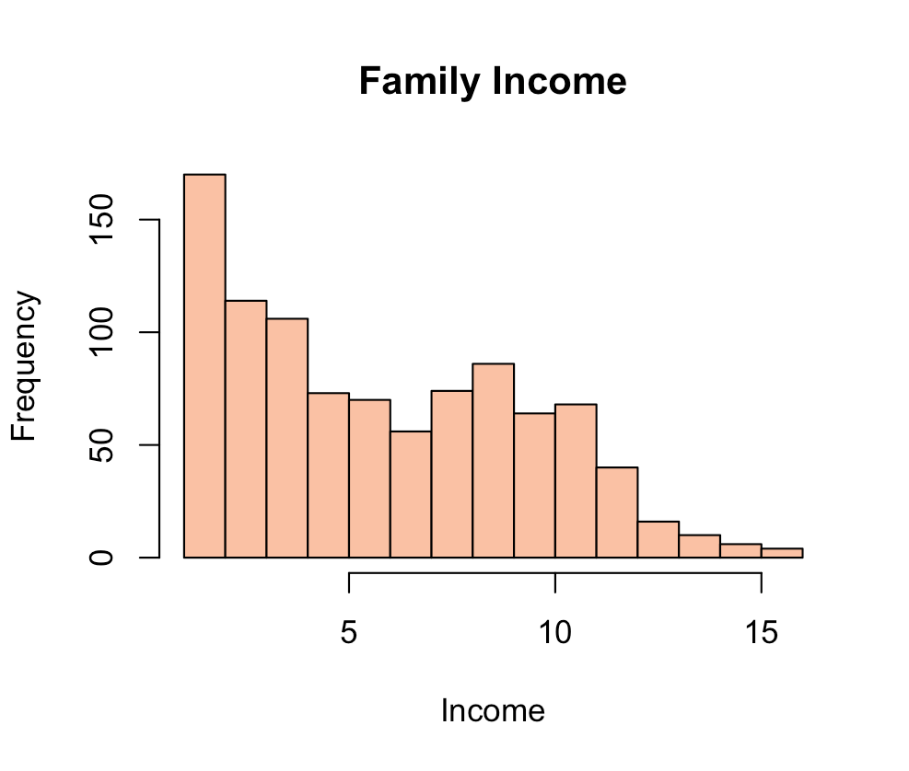


**
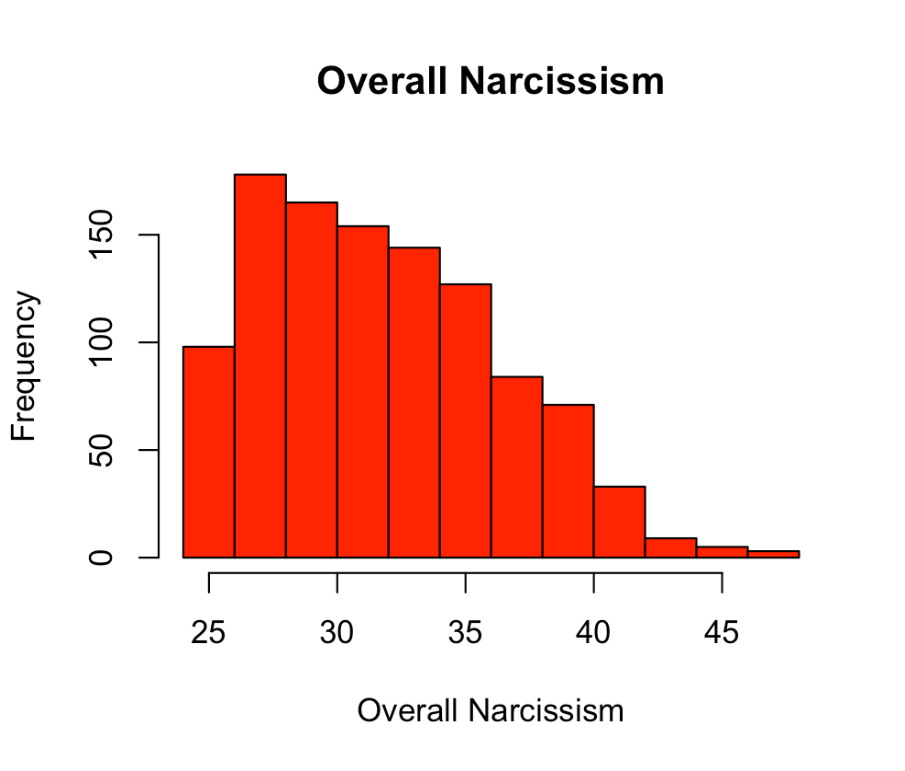
**

**
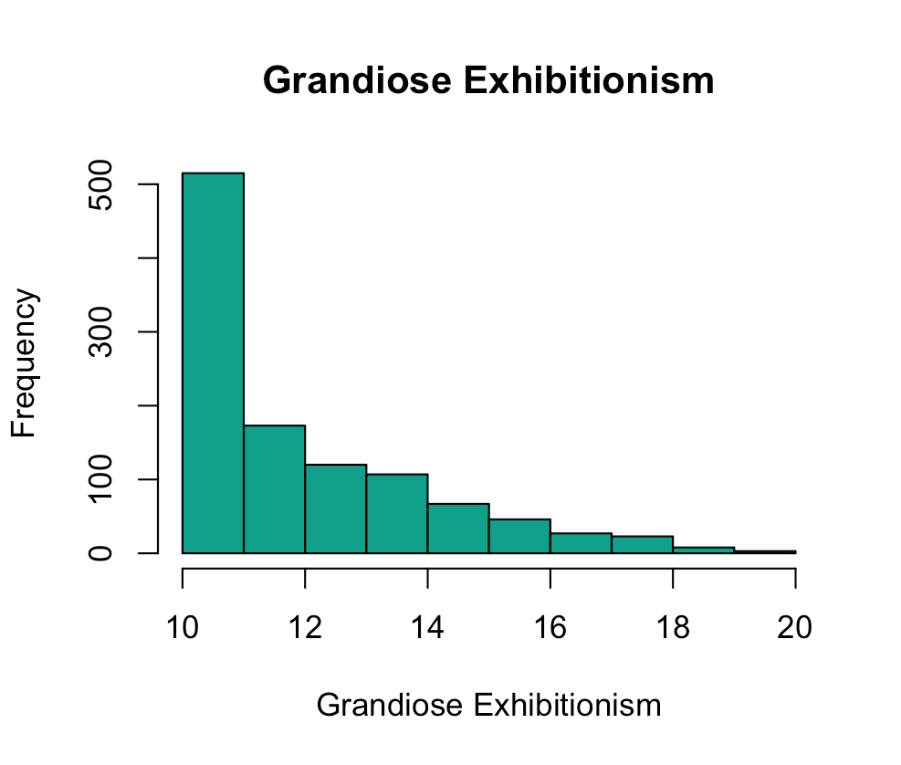
**

**
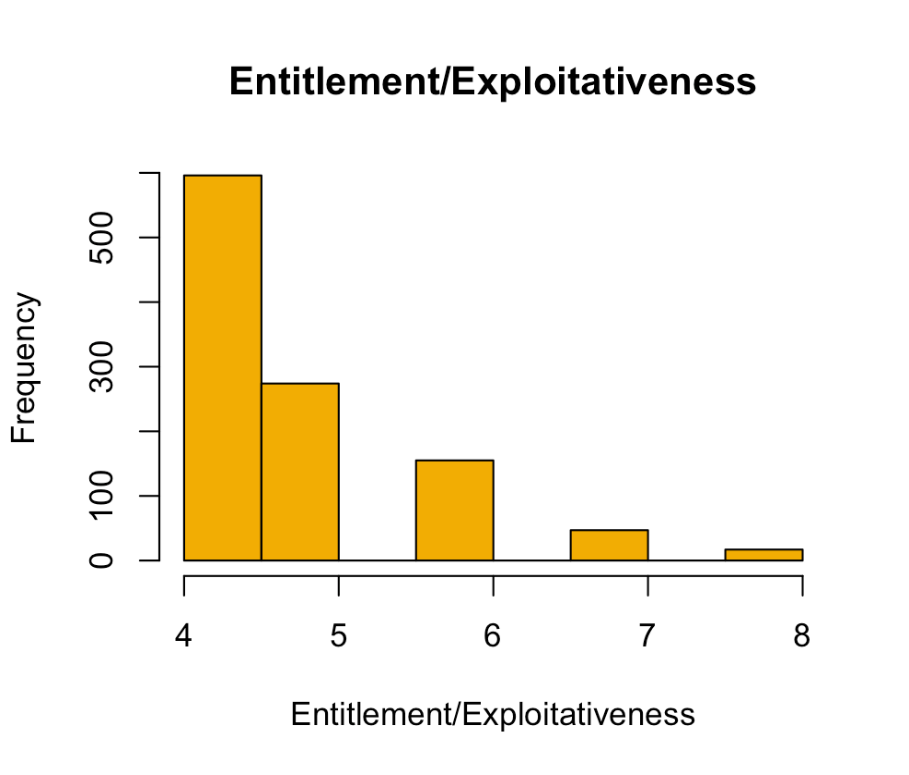
**

**
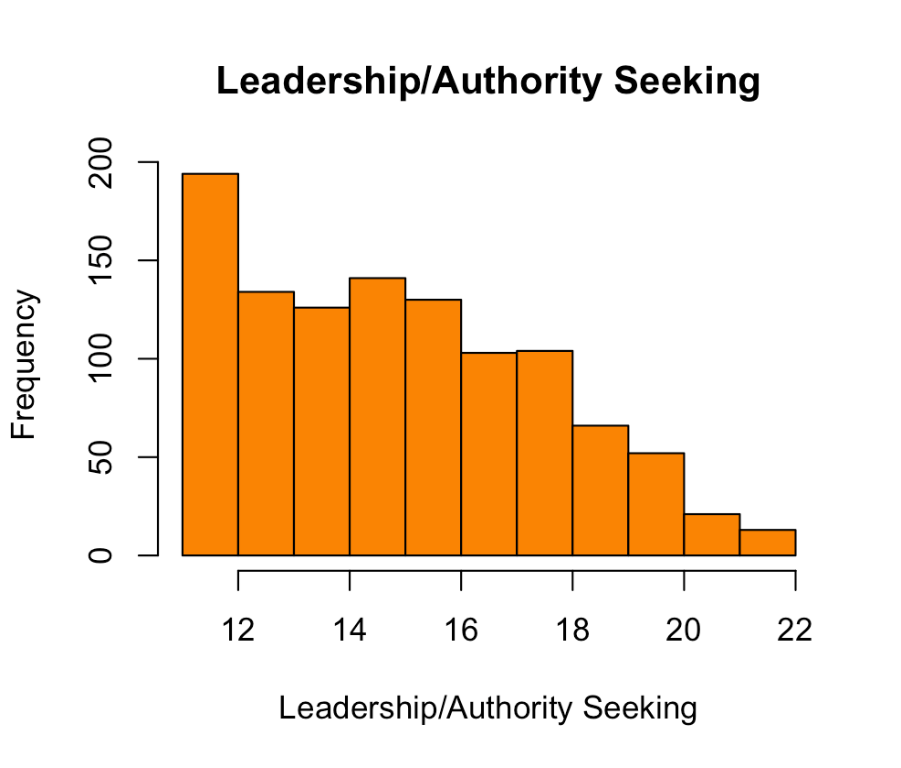
**

**SI2.4:** 2023 Raw Frequency Distributions


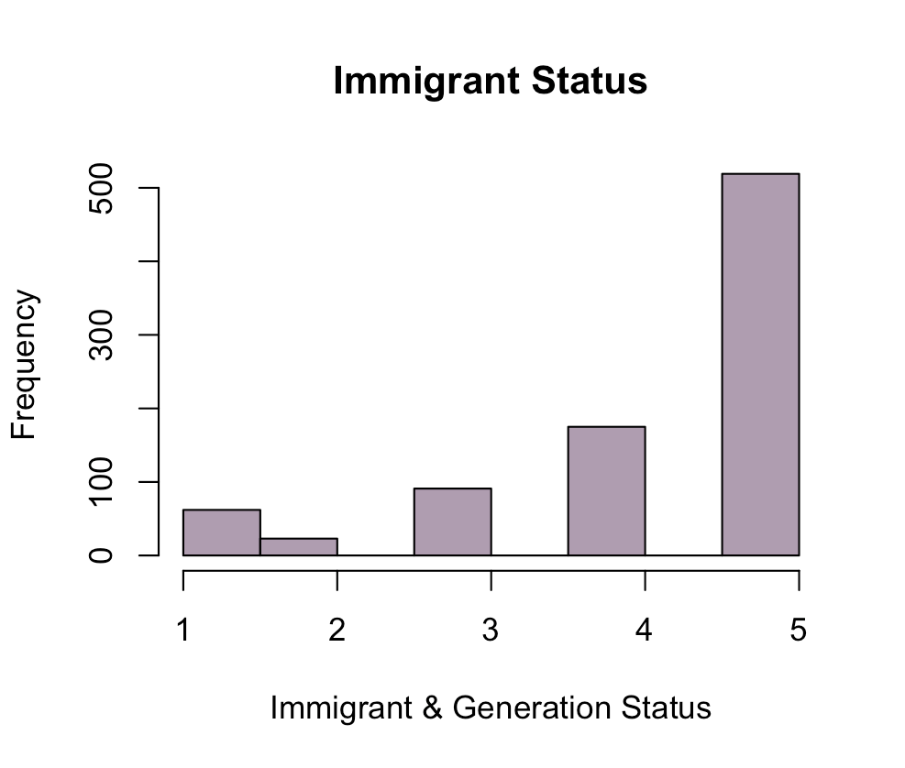


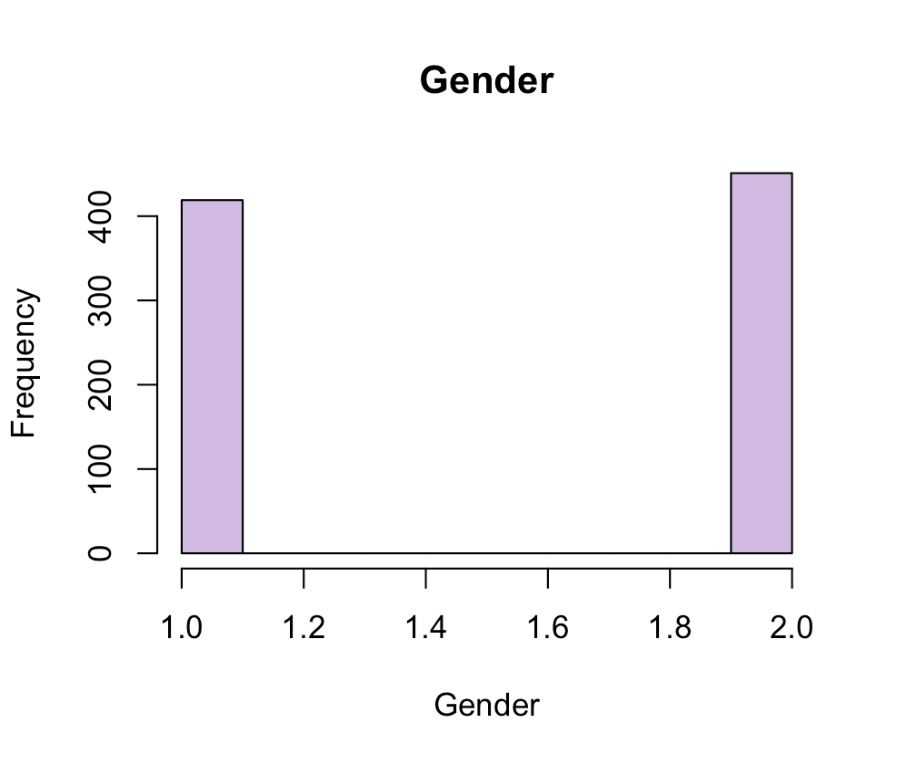


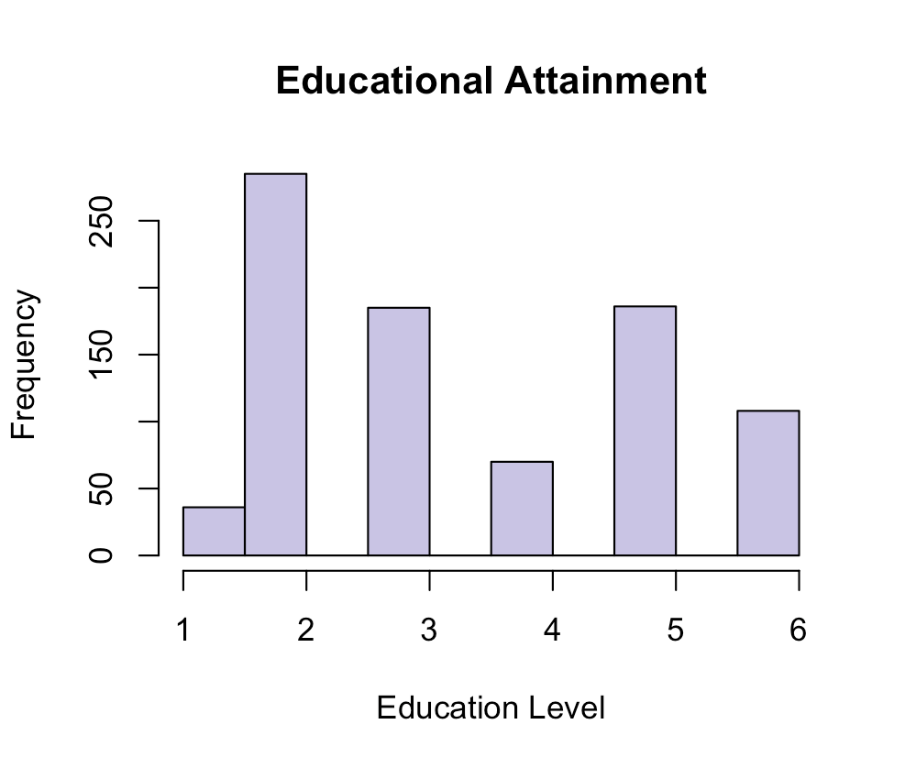


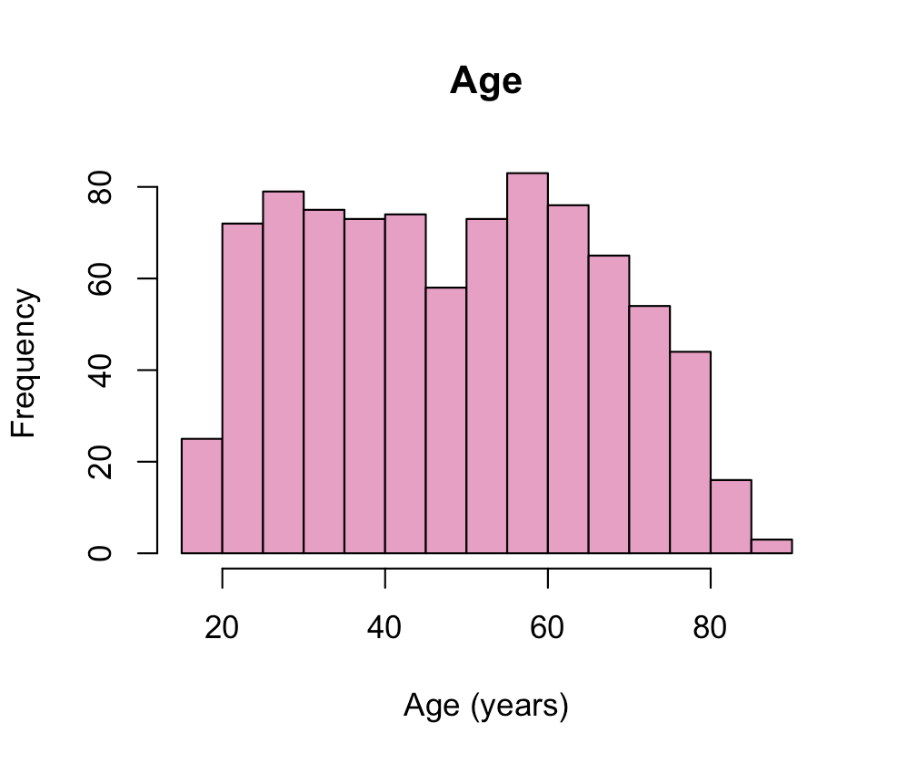


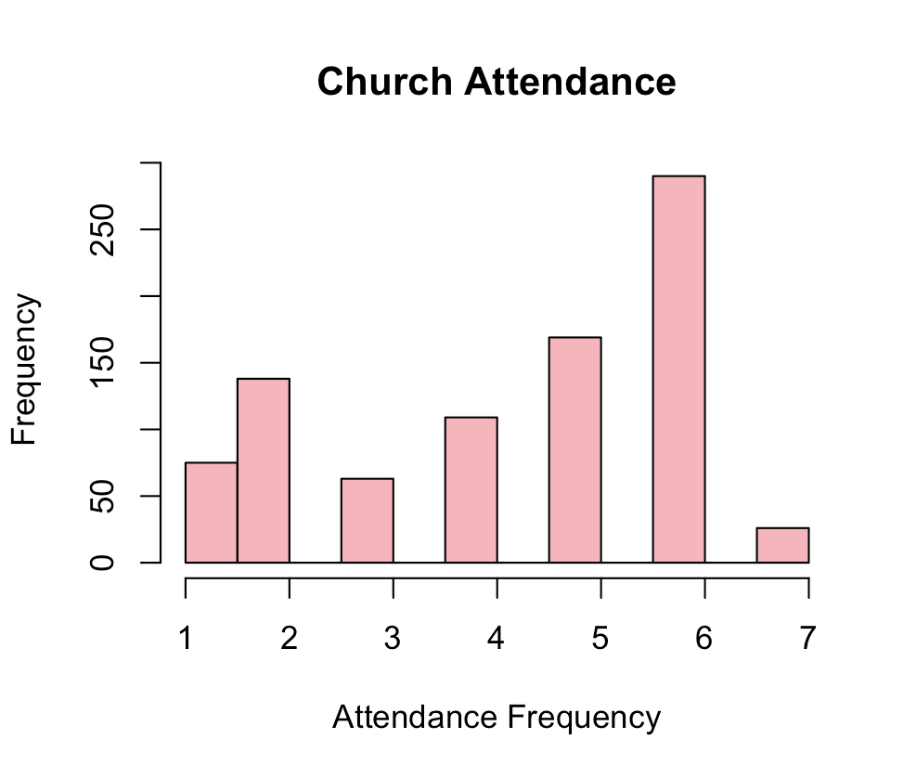


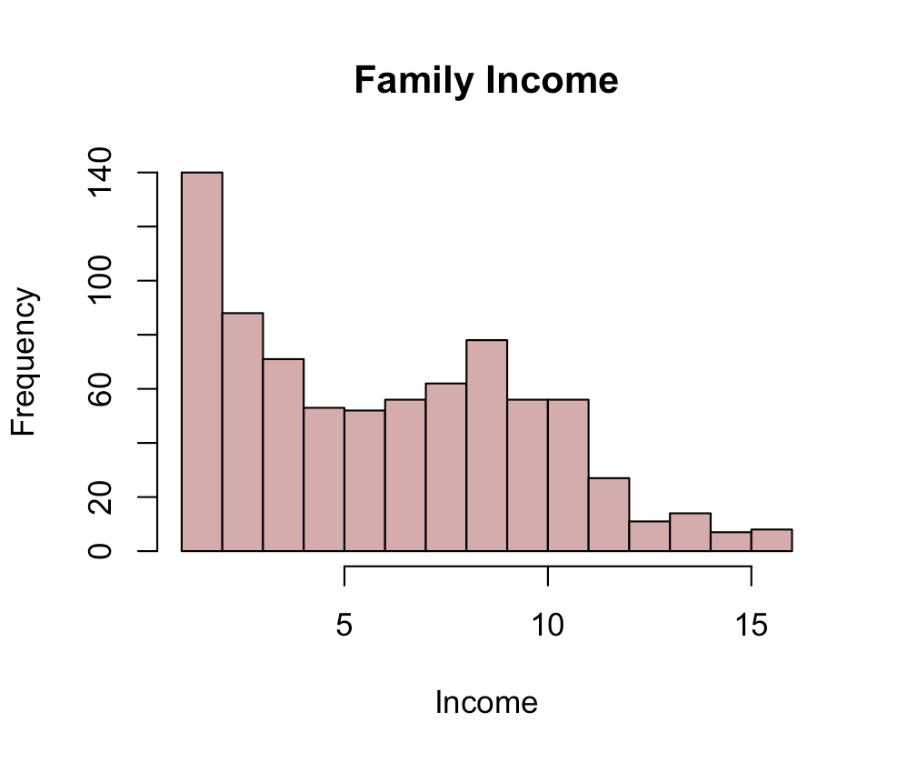


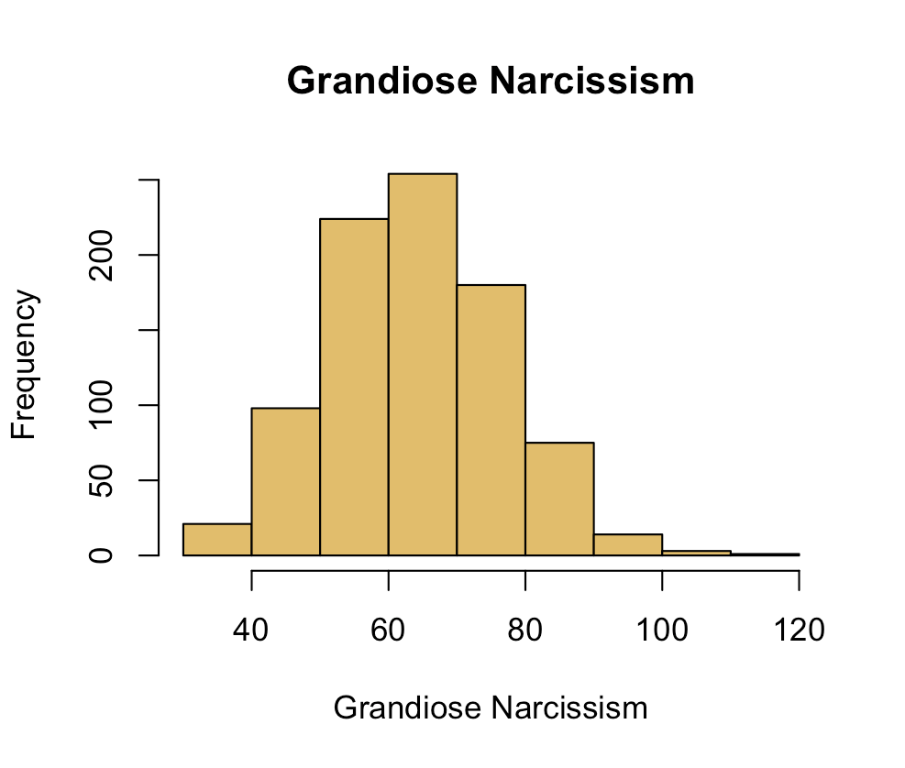


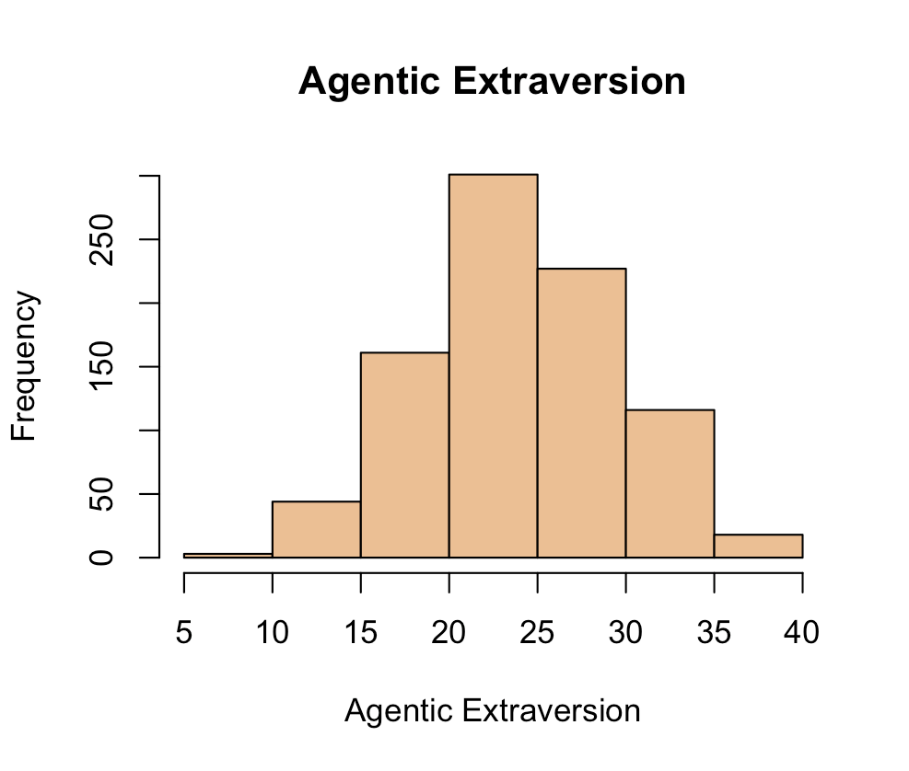


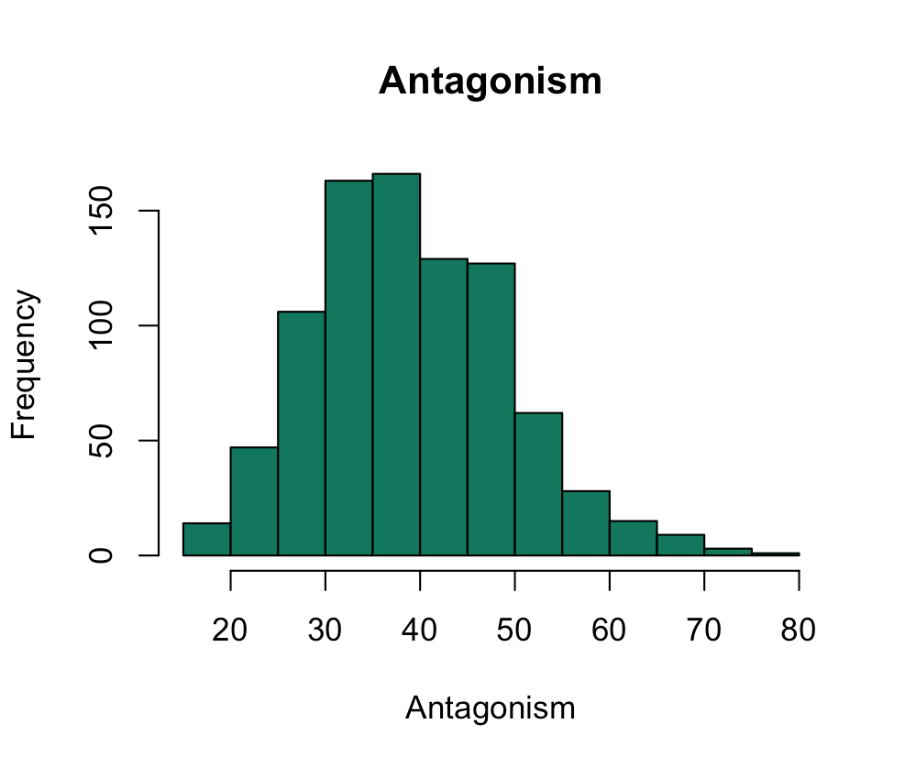


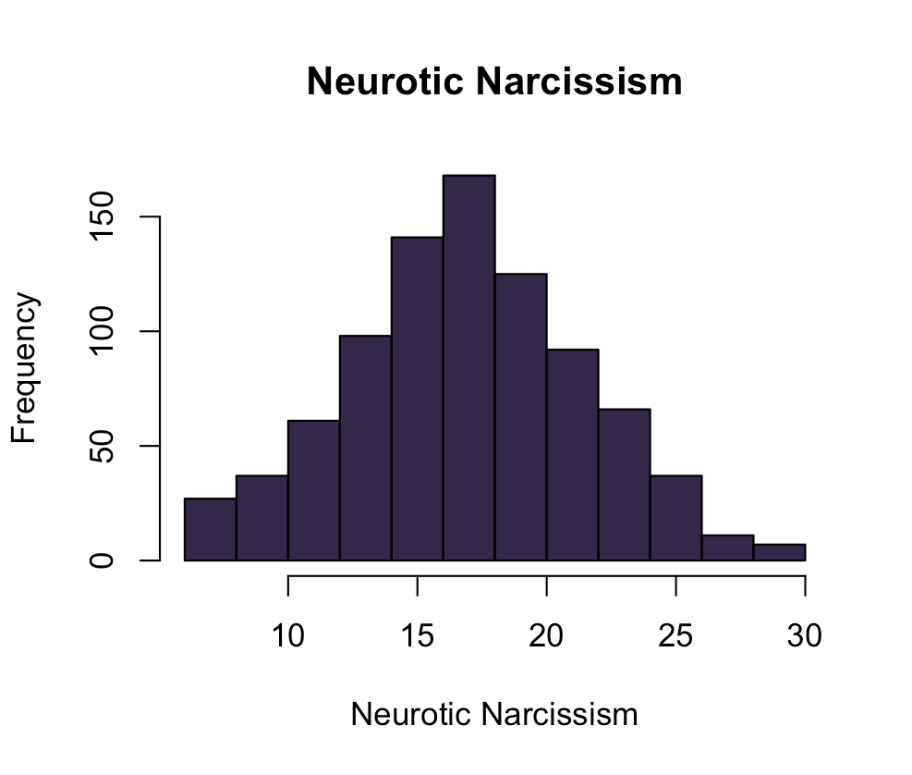


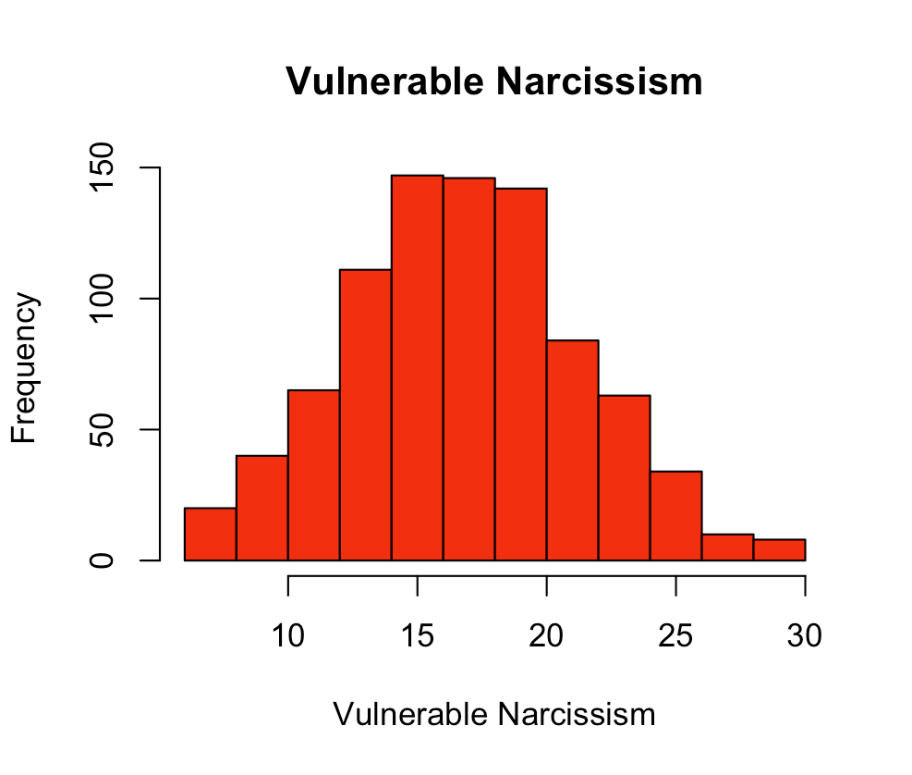


**SI3: Narcissism Composites & Reliability Scores**

**Table SI3.1:** 2021 NPI Composite Scores & Reliability

| **Factor** | **Items (alpha)** | |
| --- | --- | --- |
| Overall Narcissism | 25 (.82) | |
| Grandiose Exhibitionism | 10 (.75) | |
| Entitlement/Exploitativeness | 4 (.48) | |
| Leadership/Authority  Seeking | 11 (.74) | |
|  |  |  |

**Table SI3.2:** 2023 FFNI Composite Scores & Reliability

| **Factor** | **Items (alpha)** |
| --- | --- |
| Grandiose Narcissism | 24 (.88) |
| Agentic Extraversion | 8 (.77) |
| Antagonism | 16 (.88) |
| Neurotic Narcissism | 6 (.77) |
| Vulnerable Narcissism | 6 (.75) |

**SI4: Narcissism & Generational Status Bivariate Analyses**

**Table SI4.1:** 2021 NPI & Generation Status Correlations

| \|  \| Overall Narcissism \| Grandiose Exhibitionism \| Entitlement / Exploitativeness \| Leadership / Authority Seeking \| Generation \| \| --- \| --- \| --- \| --- \| --- \| --- \| \| Composite \|  \| 0.79** \| 0.54 \| 0.86** \| -0.18** \| \| Grandiose Exhibitionism \| 0.79** \|  \| 0.33** \| 0.42** \| -0.22** \| \| Entitlement / Exploitativeness \| 0.54** \| 0.33** \|  \| 0.29** \| -0.21** \| \| Leadership / Authority Seeking \| 0.86** \| 0.42** \| 0.29** \|  \| -0.07* \| \| Generation \| -0.18** \| -0.22** \| -0.21** \| -0.07* \|  \| \| ** p < .05, ** p <.001* \| \| \| \|  \|  \| |  |  |
| --- | --- | --- | --- | --- | --- | --- | --- | --- | --- | --- | --- | --- | --- | --- | --- | --- | --- | --- | --- | --- | --- | --- | --- | --- | --- | --- | --- | --- | --- | --- | --- | --- | --- | --- | --- | --- | --- | --- | --- | --- | --- | --- | --- | --- |

**Table SI4.2:** 2023 FFNI & Generation Status Correlations

|  | Grandiose Narcissism | Agentic Extraversion | Antagonism | Neurotic Narcissism | Vulnerable Narcissism | Generation |
| --- | --- | --- | --- | --- | --- | --- |
| Grandiose Narcissism |  | 0.80** | 0.91** | 0.28** | 0.51** | -0.21** |
| Agentic Extraversion | 0.80** |  | 0.53** | 0.17** | 0.34** | -0.19** |
| Antagonism | 0.91** | 0.53** |  | 0.17** | 0.53** | -0.18** |
| Neurotic Narcissism | 0.28** | 0.17** | 0.17** |  | 0.81** | 0.01 |
| Vulnerable Narcissism | 0.51** | 0.34** | 0.53** | 0.81** |  | -0.05 |
| Generation | -0.21** | -0.19** | -0.18** | 0.01 | -0.05 |  |
| ** p < .05, ** p <.001* | | | |  |  |  |

**SI5: Tukey HSD Models**

**Table SI5.1:** 2021 Mean Difference Generational Decreases in Narcissism

| Generational Comparisons | Immigrant vs. First | Immigrant vs. Second | Immigrant vs. Third | First vs. Second | First vs. Third | Second vs. Third |
| --- | --- | --- | --- | --- | --- | --- |
| **Overall Narcissism** | -0.04 | -0.25 | -0.26 | -0.21 | -0.22 | -0.00 |
| p-value | 0.91 | 0.00*** | 0.00*** | 0.00*** | 0.00*** | 0.99 |
| CI [95] | [-0.21, 0.12] | [-0.40, -0.10] | [-0.39, -0.12] | [-0.35, -0.07] | [-0.34, -0.09] | [-0.10, 0.09] |
| **Grandiose Exhibitionism** | -0.09 | -0.31 | -0.33 | -0.22 | -0.23 | -0.01 |
| p-value | 0.46 | 0.00*** | 0.00*** | 0.00*** | 0.00*** | 0.98 |
| CI [95] | [-0.25, 0.07] | [-0.46, -0.16] | [-0.46, -0.19] | [-0.36, -0.08] | [-0.36, -0.11] | [-0.11, 0.08] |
| **Entitlement/Exploitativeness** | -0.06 | -0.29 | -0.30 | -0.23 | -0.24 | -0.00 |
| p-value | 0.79 | 0.00*** | 0.00*** | 0.00*** | 0.00*** | 0.99 |
| CI [95] | [-0.22, 0.10] | [-0.44, -0.15] | [-0.43, -0.17] | [-0.38, -0.09] | [-0.37, -0.12] | [-0.10, 0.08] |
| **Leadership/Authority Seeking** | -0.01 | -0.10 | -0.10 | -0.09 | -0.09 | -0.00 |
| p-value | 0.99 | 0.23 | 0.15 | 0.36 | 0.25 | 1.00 |
| CI [95] | [-0.18, 0.15] | [-0.25, 0.04] | [-0.24, 0.02] | [-0.23, 0.05] | [-0.22, 0.03] | [-0.09, 0.09] |

*p < .001 ***, p < .01**, p < .05**

**Table SI5.2:** 2023 Mean Difference Generational Decreases in Narcissism

| Generational Comparisons | Immigrant vs. First | Immigrant vs. Second | Immigrant vs. Third | First vs. Second | First vs. Third | Second vs. Third |
| --- | --- | --- | --- | --- | --- | --- |
| **Grandiose Narcissism** | -0.16 | -0.29 | -0.34 | -0.13 | -0.17 | -0.04 |
| p-value | 0.11 | 0.00*** | 0.00*** | 0.15 | 0.00** | 0.71 |
| CI [95] | [-0.35, 0.02] | [-0.46, -0.13] | [-0.49, -0.19] | [-0.29, 0.02] | [-0.32, -0.03] | [-0.15, 0.06] |
| **Agentic Extraversion** | -0.09 | -0.23 | -0.28 | -0.13 | -0.19 | -0.05 |
| p-value | 0.59 | 0.00** | 0.00*** | 0.12 | 0.00** | 0.56 |
| CI [95] | [-0.28, 0.09] | [-0.39, -0.06] | [-0.43, -0.14] | [-0.30, 0.02] | [-0.33, -0.05] | [-0.16, 0.05] |
| **Antagonism** | -0.17 | -0.29 | -0.31 | -0.12 | -0.14 | -0.02 |
| p-value | 0.09 | 0.00*** | 0.00*** | 0.23 | 0.05* | 0.96 |
| CI [95] | [-0.36, 0.02] | [-0.45, -0.12] | [-0.45, -0.16] | [-0.28, 0.04] | [-0.28, 0.00] | [-0.13, 0.09] |
| **Neurotic Narcissism** | 0.09 | 0.00 | 0.04 | -0.08 | -0.05 | 0.03 |
| p-value | 0.60 | 0.99 | 0.89 | 0.55 | 0.80 | 0.87 |
| CI [95] | [-0.10, 0.28] | [-0.16, 0.17] | [-0.10, 0.19] | [-0.25, 0.08] | [-0.19, 0.09] | [-0.07, 0.14] |
| **Vulnerable Narcissism** | 0.05 | -0.10 | -0.05 | -0.15 | -0.10 | 0.04 |
| p-value | 0.88 | 0.42 | 0.80 | 0.07 | 0.23 | 0.69 |
| CI [95] | [-.13, 0.24] | [-0.27, 0.06] | [-0.20, 0.09] | [-0.32, 0.01] | [-0.25, 0.03] | [-0.06, 0.15] |

*p < .001 ***, p < .01**, p < .05**

**SI6: Narcissism & Generational Status Regression Analyses**

**Table SI6.1:** 2021 Generational Status as Predictor of NPI Narcissism Factors

|  | Overall Narcissism | Grandiose Exhibitionism | Entitlement / Exploitativeness | Leadership / Authority Seeking |
| --- | --- | --- | --- | --- |
| Generation | -0.07*** | -0.08*** | -0.08*** | -0.03* |
|  | (0.01) | (0.01) | (0.01) | (0.01) |
| Age | -0.00*** | -0.00*** | -0.00*** | -0.00* |
|  | (0.00) | (0.00) | (0.00) | (0.00) |
| Male | 0.09** | 0.04 | 0.10*** | 0.09** |
|  | (0.03) | (0.03) | (0.03) | (0.03) |
| Education | 0.00 | -0.00 | -0.00 | 0.01 |
|  | (0.01) | (0.01) | (0.01) | (0.01) |
| Family Income | 0.01* | 0.00 | 0.00 | 0.01** |
|  | (0.00) | (0.00) | (0.00) | (0.00) |
| Church Attendance | 0.03*** | 0.03*** | 0.02** | 0.03*** |
|  | (0.00) | (0.00) | (0.00) | (0.00) |
| Constant | 0.28 | 0.46 | 0.53 | -0.06 |
| *p < .001 ***,*  *p < .01**,*  *p < .05** | R² [0.11], F(6, 898) = 18.82, p <.001 | R² [0.11], F(6, 913) = 19.87, p <.001 | R² [0.13], F(6, 914) = 24.77, p <.001 | R² [0.05], F(6, 909) = 8.75, p <.001 |

**Table SI6.2:** 2023 Generational Status as Predictor of FFNI Narcissism Factors

|  | Grandiose Narcissism | Agentic Extraversion | Antagonism | Neurotic Narcissism | Vulnerable Narcissism |
| --- | --- | --- | --- | --- | --- |
| Generation | -0.07*** | -0.06*** | -0.06*** | 0.03 | 0.00 |
|  | (0.01) | (0.01) | (0.01) | (0.01) | (0.01) |
| Age | -0.01*** | -0.00*** | -0.01*** | -0.00*** | -0.01*** |
|  | (0.00) | (0.00) | (0.00) | (0.00) | (0.00) |
| Male | 0.16*** | 0.11*** | 0.15*** | -0.08** | -0.05 |
|  | (0.03) | (0.03) | (0.03) | (0.03) | (0.03) |
| Education | 0.00 | 0.01 | -0.01 | 0.04*** | 0.01 |
|  | (0.01) | (0.01) | (0.01) | (0.01) | (0.01) |
| Family Income | -0.00 | 0.00 | -0.00 | -0.00 | -0.00 |
|  | (0.00) | (0.00) | (0.00) | (0.00) | (0.00) |
| Church Attendance | 0.02** | 0.03*** | 0.01 | 0.00 | 0.01 |
|  | (0.00) | (0.00) | (0.00) | (0.01) | (0.00) |
| Constant | 0.62 | 0.38 | 0.71 | 0.14 | 0.49 |
| *p < .001 ***,*  *p < .01**,*  *p < .05** | R² [0.21], F(6, 756) = 34.52, p <.001 | R² [0.16], F(6, 756) = 24.03, p <.001 | R² [0.20], F(6, 756) = 31.91, p <.001 | R² [0.07], F(6, 756) = 9.82, p <.001 | R² [0.13], F(6, 756) = 20.10, p <.001 |
